# Somatic genome editing with CRISPR/Cas9 generates and corrects a metabolic disease

**DOI:** 10.1038/srep44624

**Published:** 2017-03-16

**Authors:** Kelsey E. Jarrett, Ciaran M. Lee, Yi-Hsien Yeh, Rachel H. Hsu, Rajat Gupta, Min Zhang, Perla J. Rodriguez, Chang Seok Lee, Baiba K. Gillard, Karl-Dimiter Bissig, Henry J. Pownall, James F. Martin, Gang Bao, William R. Lagor

**Affiliations:** 1 Department of Molecular Physiology and Biophysics, Baylor College of Medicine, Houston, TX 77030, USA; 2 Integrative Molecular and Biomedical Sciences Graduate Program, Baylor College of Medicine, Houston, TX 77030, USA; 3 Department of Bioengineering, Rice University, Houston, TX 77030, USA; 4 Houston Methodist Research Institute, Houston, TX 77030, USA; 5 Weill Cornell Medicine, Houston, TX 77030, USA; 6 Center for Cell and Gene Therapy, Department of Molecular and Cellular Biology, Baylor College of Medicine, Houston, Texas 77030, USA; 7 Texas Heart Institute, Houston, TX 77030, USA

## Abstract

Germline manipulation using CRISPR/Cas9 genome editing has dramatically accelerated the generation of new mouse models. Nonetheless, many metabolic disease models still depend upon laborious germline targeting, and are further complicated by the need to avoid developmental phenotypes. We sought to address these experimental limitations by generating somatic mutations in the adult liver using CRISPR/Cas9, as a new strategy to model metabolic disorders. As proof-of-principle, we targeted the low-density lipoprotein receptor (*Ldlr*), which when deleted, leads to severe hypercholesterolemia and atherosclerosis. Here we show that hepatic disruption of *Ldlr* with AAV-CRISPR results in severe hypercholesterolemia and atherosclerosis. We further demonstrate that co-disruption of *Apob*, whose germline loss is embryonically lethal, completely prevented disease through compensatory inhibition of hepatic LDL production. This new concept of metabolic disease modeling by somatic genome editing could be applied to many other systemic as well as liver-restricted disorders which are difficult to study by germline manipulation.

Metabolic disease modeling is an essential component of biomedical research and a mandatory prerequisite for the treatment of human disease. Genetically engineered mice are the most frequently used model organism to study metabolic liver diseases, and have been invaluable in advancing our knowledge of many human disorders. However, many metabolic genes also play important roles in development, and their loss may result in embryonic lethality. The mouse genome informatics (MGI) database describes that at least 30% of all homozygous mutant lines created are embryonic or perinatal lethal^1^. Specific examples include methylmalonic aciduria (*Mmachc*)^2^, mevalonic aciduria (*Mvk*)^3^, lipoprotein lipase deficiency (*Lpl*)^4^, and hypobetalipoproteinemia (*Apob*)^5^, which underlie devastating human liver diseases. In some cases, hypomorphic alleles have been developed that recapitulate key aspects of a phenotype, such as ornithine transcarbamylase (*Otc*) deficiency, where the *spf* mutant has 10%, and the *Spf*^*ash*^ mutant 5%, of normal liver enzymatic activity^6^. Nevertheless, establishing a hypomorphic mouse model is difficult and often does not fully recapitulate the human disease.

Several methods exist to study liver-expressed metabolic genes in adult mice. The cleanest of these are conditional mouse models, where the gene of interest is deleted by a liver-specific CRE recombinase transgene or on demand with viral delivery of CRE. Although robust, this technology is time-consuming and not well suited to testing multiple candidate genes, either alone or in combination. Loss-of-function studies can also be performed through the use of chemically modified antisense oligonucleotides (ASOs)^7^, locked nucleic acids^8^, siRNA nanoparticles^9^, and viral expression of shRNA^10^. These methods have advantages for achieving acute knockdown of target genes, but all have unique caveats, and leave varying degrees of residual expression and activity. Recent advances suggest that somatic genome editing could be used for genetic loss-of-function in adult animals.

Clustered Regularly-Interspaced Short Palindromic Repeats/Cas9 (CRISPR/Cas9) is a bacterial immune system that has been adapted for genome editing in mammalian cells^11^,^12^. Cas9 is a programmable nuclease that generates double-stranded breaks (DSB) in DNA dictated by binding of a ~20 nucleotide recombinant “guide RNA” (gRNA) to the target site. DSB’s produced by Cas9 are most often repaired through the cell’s error-prone non-homologous end joining (NHEJ) pathway, resulting in small insertions or deletions (indels). The vast majority of indels shift the reading frame^13^, introducing a premature stop codon or resulting in nonsense mediated decay of the mRNA - effectively “knocking out” a gene. We and others have shown that delivery of CRISPR/Cas9 can effectively knockout liver-expressed genes through methods such as hydrodynamic tail vein injection^14^ and lipid nanoparticles^15^. Viral vectors offer the advantage of near complete liver transduction, eliminating potential redundancy from neighboring hepatocytes. Adenoviral vectors have been used to deliver CRISPR/Cas9 to disrupt *Pcsk9*^16^ in mouse liver. Adeno-Associated Viral (AAV) vectors have also been used for somatic genome editing in adult mouse liver^17^,^18^, including the identification of a smaller Cas9 ortholog from *Staphylococcus aureus* (SaCas9) that can edit genes with high specificity^18^. Collectively, these studies suggest that CRISPR/Cas9 genome editing is very efficient in murine hepatocytes. Somatic genome editing using CRISPR/Cas9 might be used to establish novel metabolic disease models. Hypercholesterolemia and associated atherosclerosis is a major problem in the developed world, and would serve as an excellent test case for modeling a common metabolic disease.

The liver secretes triglyceride-rich very low density lipoprotein (VLDL) particles into the circulation to provide energy to the rest of the body. These particles each contain one Apolipoprotein B (APOB) molecule as their core structural component, an essential player in export of excess triglyceride from the liver. VLDL particles are broken down by lipases in the circulation, generating smaller, cholesterol-enriched, low density lipoprotein (LDL) particles. Excess LDL is normally removed through the action of the low density lipoprotein receptor (LDLR), which is highly expressed in the liver, where it is believed to account for approximately 70% of LDL clearance^19^,^20^. Mutations in the *LDLR* gene are the basis of Familial Hypercholesterolemia (FH) (MIM 143890), a Mendelian lipid disorder characterized by exceptionally high levels of LDL cholesterol (LDL-C), xanthomas, and severe atherosclerotic vascular disease. We previously showed that human FH could be modeled in chimeric mice through transplantation with hepatocytes from an FH patient, and the ensuing hypercholesterolemia corrected by additive gene therapy with AAV9 vectors^21^.

Here we generate the first model of metabolic liver disease via somatic genome editing. Mice treated with AAV-CRISPR vectors to somatically disrupt the *Ldlr* gene in the liver develop a severe hypercholesterolemia as well as a systemic metabolic phenotype- atherosclerosis. Concomitant disruption of *Apob* is protective from hypercholesterolemia and atherosclerosis, and demonstrates the utility of somatic genome editing to validate potential therapeutic targets for metabolic liver disorders.

## Results

### Somatic disruption of metabolic genes with AAV-CRISPR

**Figure 1:**
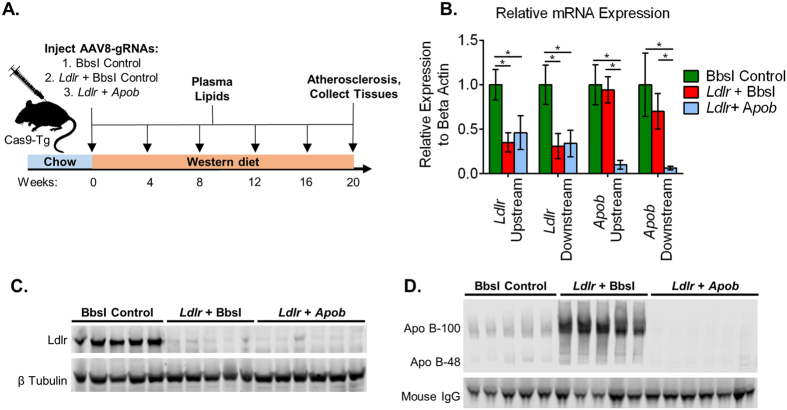
Somatic disruption of *Ldlr* and *Apob*. **(A)** Experimental design- Male C57BL/6 J mice were injected with a total of 6 × 10^11^ genome copies of AAV, placed on Western Diet, and followed for 20 weeks. (**B**) Expression of mRNA *Ldlr* and *Apob* in the liver using primers upstream and downstream of the gRNA site. (**C**) LDLR and beta tubulin Western blot in mouse liver. (**D**) Apo B protein levels in plasma. Uncropped Western blot images are shown in Supplementary Data. For all panels: BbsI control *n* = 5, *Ldlr* gRNA + BbsI *n* = 5, *Ldlr* + *Apob* gRNA *n* = 6; data are represented as mean +/− S.D. and *p < 0.05.

Small guide RNAs were designed using E-CRISP (http://www.e-crisp.org) targeting either exon 14 of *Ldlr* or exon 5 of *Apob*. The gRNAs were cloned into our AAV vector that expresses the gRNA from the U6 promoter, upstream of green fluorescent reporter driven by the ubiquitous chicken beta (CB) actin promoter. Male Cas9 transgenic mice^22^ (6–9 weeks old) were injected with 6 × 10^11^ genome copies (GC) of AAV8 encoding the different gRNA’s. Mice were randomly assigned one of three groups: (*i*) BbsI (empty cloning site as a “nontargeting” control, 6 × 10^11^ GC), (*ii) Ldlr* gRNA + BbsI (3 × 10^11^ each), and (*iii) Ldlr* gRNA + *Apob* gRNA (3 × 10^11^ each). The animals were placed on Western diet, a high fat cholesterol-rich diet, followed for 20 weeks, and sacrificed to assess atherosclerosis, plasma cholesterol, Ldlr and Apo B protein levels, and editing efficiency (Fig. 1A). Mice in groups receiving a gRNA to *Ldlr* had greater than 50% decreases in *Ldlr* mRNA expression (Fig. 1B). Animals receiving the *Apob* gRNA showed more than 90% knockdown of *Apob* mRNA (Fig. 1B). For both the *Ldlr* and *Apob* transcripts, similar results were obtained using primers upstream and downstream of the cut site. LDLR protein was readily detectable in livers from the BbsI control group, and nearly completely absent from the *Ldlr* gRNA and *Ldlr* + *Apob* gRNA groups (Fig. 1C). The *Ldlr* gRNA group had a dramatic increase in Apo B-100 and Apo B-48 compared to the control animals, while the *Ldlr* + *Apob* gRNA treated mice had a nearly complete loss of Apo B protein (Fig. 1D).

### Efficiency and specificity of somatic genome editing

**Figure 2:**
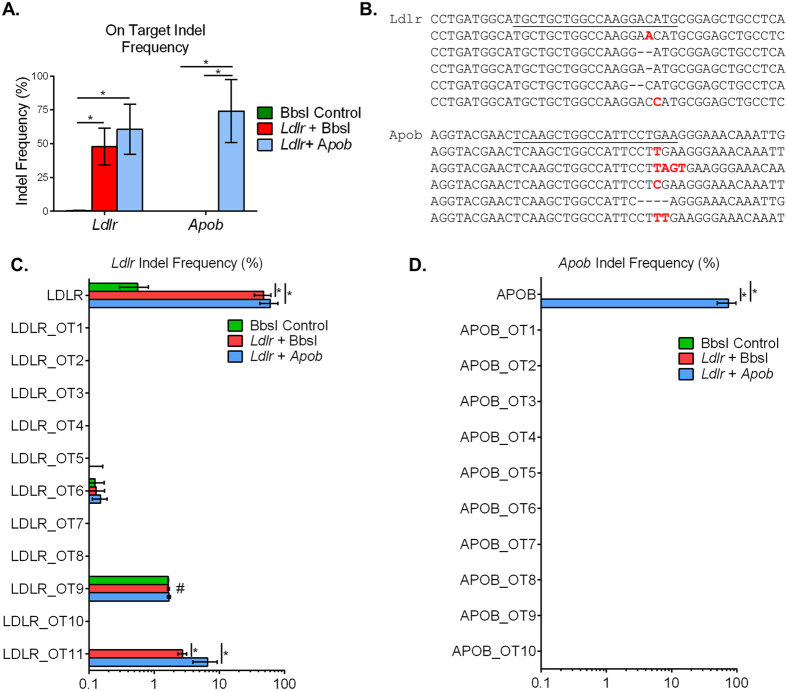
Deep sequencing of on- and off-target editing. (**A**) Indel frequency at *Ldlr* and *Apob* in mouse liver. (**B**) Most common indels observed at each target locus. Insertions are highlighted in red and deletions are shown with dashed lines. (**C**) Indel frequency at *Ldlr* and the top ranked off-target sites. (**D**) Indel frequency for A*pob* and the top ranked off-target sites. Data represent *n* = 4 for each group, and are reported as mean +/− S.D. with *p < 0.05; ^#^denotes a repetitive amplicon resulting in sequencing error.

**Figure 3:**
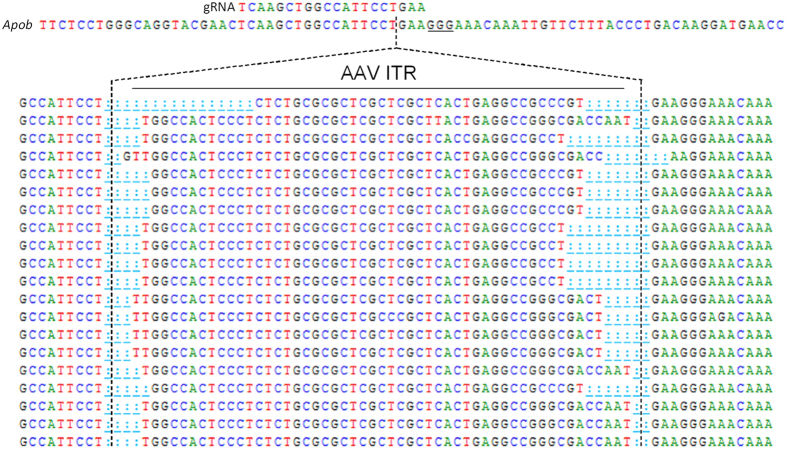
Insertion of AAV-ITR sequences at a CRISPR/Cas9 target site. AAV ITR insertions at the *Apob* target site. Short ITR sequences were detected at the gRNA cut site 3 bases upstream of the PAM (underlined) in all mice receiving the *Apob* gRNA. Representative sequencing reads are shown.

The efficiency and specificity of editing was assessed by deep sequencing of livers following the 20 week study. In addition to the target regions of *Ldlr* and *Apob*, we used the COSMID algorithm^23^ to predict potential off-target sites in the mouse genome. On- and off-target sites were amplified by PCR to generate barcoded libraries (n = 4 per group) (Supplementary Table 1). Deep sequencing revealed a high degree of editing in the *Ldlr* gene in both groups receiving the *Ldlr* gRNA with a mean indel formation of 54.3 ± 16.6% (Fig. 2A). In the case of *Apob*, the group receiving a gRNA targeting this gene had an impressive rate of indel formation of 74.1 ± 23.4% (Fig. 2A). The most common mutations were small insertions and deletions occurring around positions −3 to −5 relative to the Protospacer Adjacent Motif (PAM) (Fig. 2B). One of the predicted off-target sites for the *Ldlr* gene (OT 11) had a low frequency of mutations which was detectable above background at 5.12 ± 2.02% (Fig. 2C). This particular off-target site is within an intron of the *Stx8* gene. No detectable off-target editing was detected for any predicted sites for the *Apob* gRNA (Fig. 2D). However, indel formation was observed at several highly similar predicted sites in mice that received the BbsI stuffer sequence present in many CRISPR plasmids^24^, ranging from 1.3–30% depending on location (Supplementary Table 2). Perhaps most interestingly, small insertion events containing sequences from the AAV inverted terminal repeats (ITRs) were detected at the cut site with the highest editing efficiency (exon 5 of the *Apob* gene), accounting for 10–26% of the total reads (Fig. 3).

### Metabolic disease modeling and correction with CRISPR/Cas9

**Figure 4:**
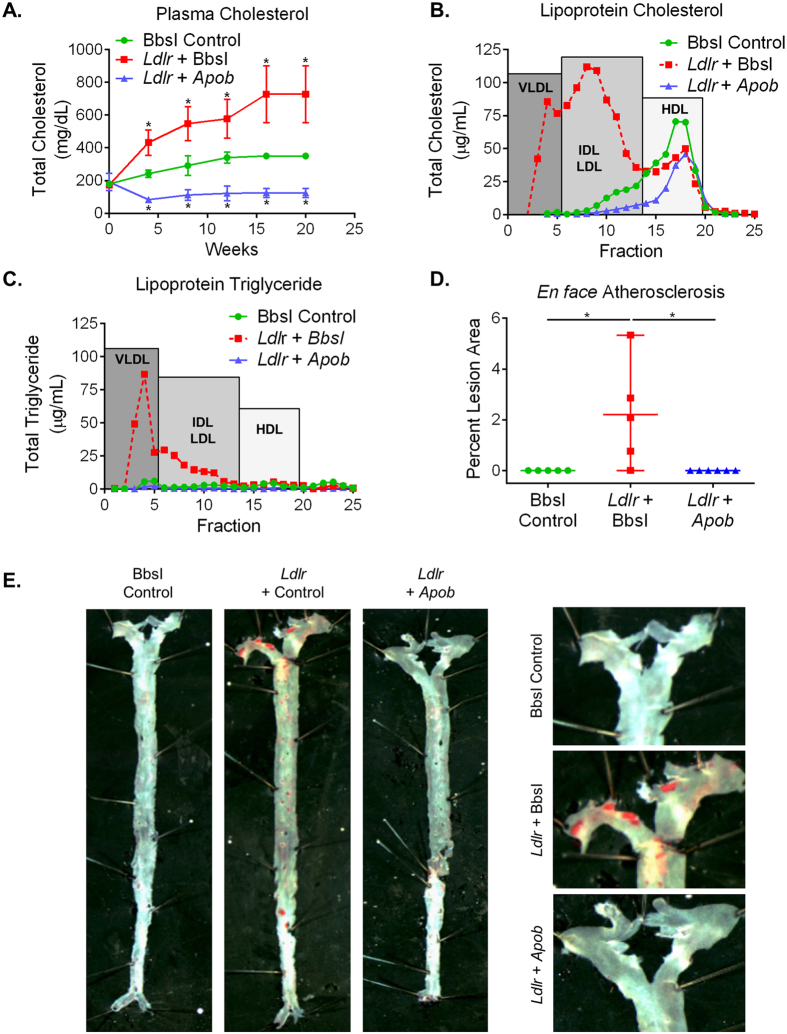
Physiological effects of somatic disruption of Ldlr and *Apob*. (**A**) Fasted (5 hr) plasma cholesterol levels over time following AAV-CRISPR. (**B**) Lipoprotein cholesterol levels in plasma pooled from all mice in each group separated by size exclusion chromatography. (**C**) Lipoprotein triglyceride levels in plasma pooled from all mice in each group separated by size exclusion chromatography. (**D**) Quantification of atherosclerotic lesion areas in the aortae. (**E**) *En face* atherosclerotic lesions in the aortic arch stained with Oil Red O. For all panels: BbsI control *n* = 5, *Ldlr* gRNA + BbsI *n* = 5, *Ldlr* + *Apob* gRNA *n* = 6; data are represented as mean +/− S.D. and *p < 0.05.

Mice receiving the control virus displayed a gradual rise in plasma cholesterol from the Western diet, reaching a maximum of 350 ± 18.7 mg/dL after 20 weeks. The *Ldlr* gRNA group had significantly higher plasma cholesterol which increased to a maximum of 728 ± 174 mg/dL. In contrast, mice receiving both the *Ldlr* and *Apob* gRNAs exhibited a rapid drop in plasma cholesterol which remained low for the duration of the 20 weeks, with a final concentration of 125 ± 27.3 mg/dL (Fig. 4A). The majority of the cholesterol in the mice receiving the *Ldlr* gRNA was carried in the VLDL and IDL/LDL fractions. In contrast, concomitant disruption of *Apob* caused a dramatic reduction in cholesterol in the VLDL and IDL/LDL fractions, with a further lowering of HDL (Fig. 4B). Triglyceride levels were markedly increased in the VLDL and IDL/LDL fractions from mice in the *Ldlr* + BbsI group, but not in control animals or those with *Apob* disruption (Fig. 4C). Plasma free fatty acid levels were slightly lower in the *Ldlr* + *Apob* group at baseline, and remained low during 20 weeks on the Western Diet, in contrast to the slight increase in the control and *Ldlr* gRNA groups (Supplementary Figure 1). Atherosclerotic lesions were readily visible in whole aortae of the mice receiving the *Ldlr* gRNA with a total lesion area of 2.2% ± 2.1% (Fig. 4D,E). The control mice had no detectable lesion as expected for C57BL/6 J mice on this diet. Interestingly, disruption of *Apob* was atheroprotective in mice that received *Ldlr* + *Apob* gRNAs (Fig. 4D,E) despite the near complete absence of LDLR protein in the livers of these animals (Fig. 1C).

**Figure 5:**
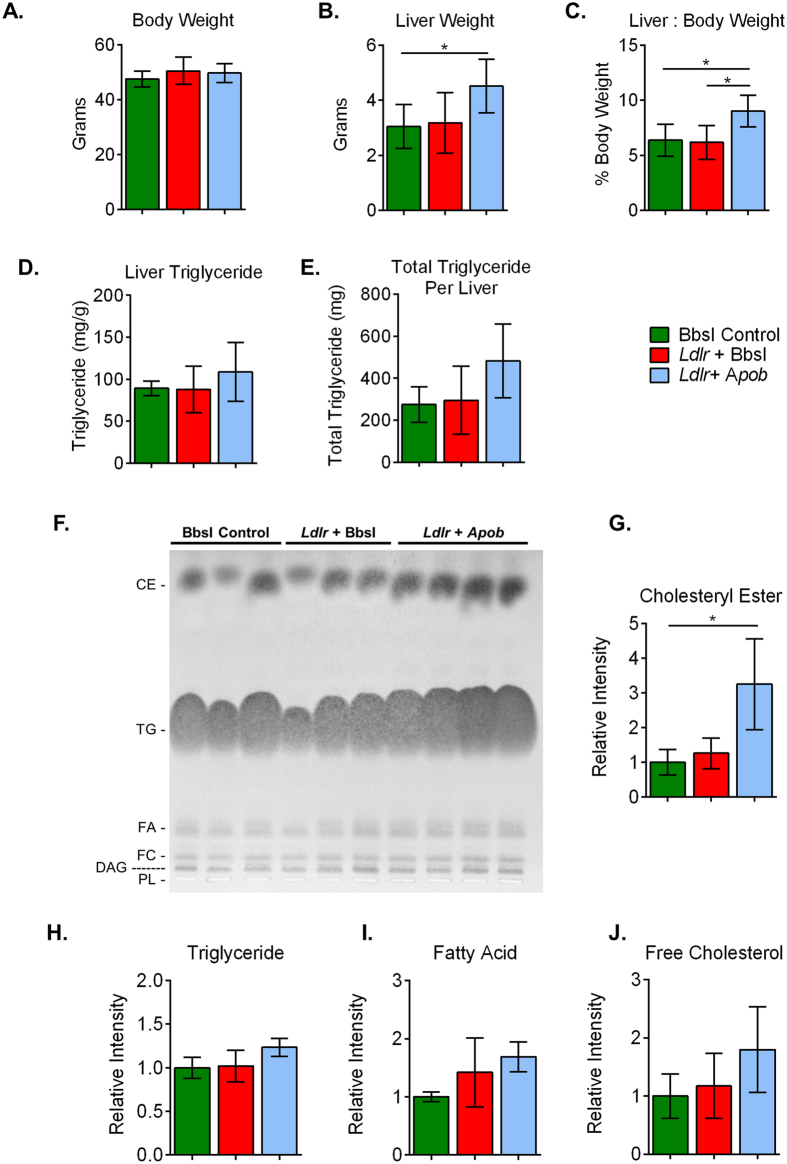
Impact of *Ldlr* and *Apob* disruption on liver lipids. (**A**) Body weight after 20 weeks on Western diet. (**B**) Liver weight. (**C**) Ratio of liver to body weight. (**D**) Triglyceride content per gram of liver. (**E**) Triglyceride content per total liver. (**F**) Representative thin layer chromatography image showing cholesteryl ester (CE), triglyceride (TG), free fatty acid (FA), free cholesterol (FC), diacylglyceride (DAG), and phospholipid (PL). Quantitation of liver lipids based on densitometry of TLC plates (**G**–**J**). For (**A**–**E**): BbsI control *n* = 5, *Ldlr* gRNA + BbsI *n* = 5, *Ldlr* + *Apob* gRNA *n* = 6; data are represented as mean +/− S.D. and *p < 0.05. For (**F**): BbsI control *n* = 3, *Ldlr* gRNA + BbsI *n* = 3, *Ldlr* + *Apob* gRNA *n* = 4.

Body weights were comparable between all groups (Fig. 5A), but mice that received the *Ldlr* + *Apob* gRNAs had substantially higher liver weights. (Fig. 5B,C). Livers from these animals were buoyant in aqueous formalin solution (Supplementary Figure 2), visibly fatty, and trended towards higher total triglyceride content when measured enzymatically (Fig. 5D,E). To further investigate lipid content, total lipids were extracted by the Bligh-Dyer method^25^ and separated by thin layer chromatography (Fig. 5F). Cholesteryl ester levels were significantly higher in the *Ldlr* + *Apob* group (Fig. 5G), and triglycerides trended toward an increase (Fig. 5H). There were no significant differences between groups in free fatty acid or free cholesterol levels (Fig. 5I,J).

**Figure 6:**
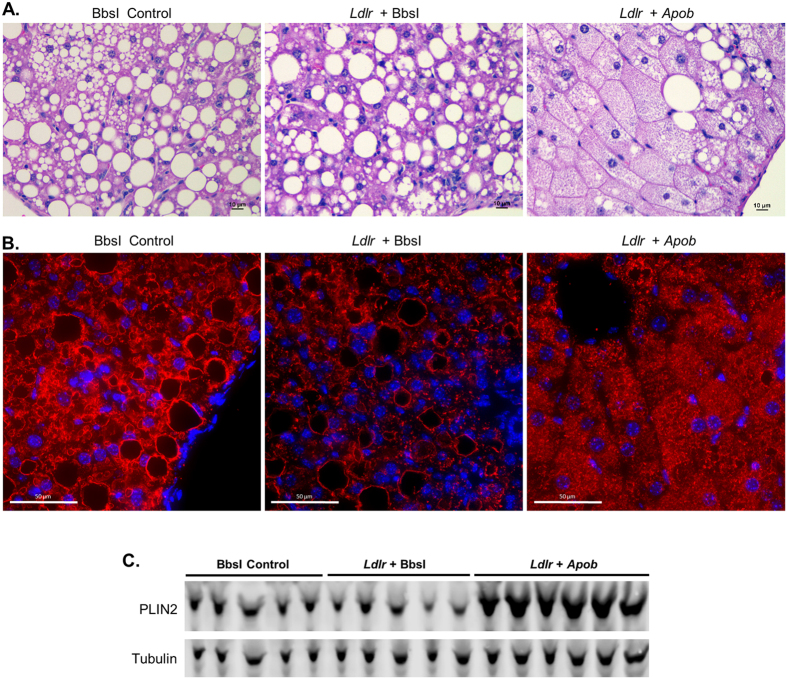
Concomitant disruption of *Ldlr* and *Apob* results in microvesicular steatosis. Liver sections from mice injected with *BbsI* control, *Ldlr* gRNA or *Ldlr* + *Apob* gRNA vectors were analyzed at 20 weeks after treatment. (**A**) H & E staining of livers at 400X magnification. (**B**) Perilipin 2 (red) immunohistochemistry and DAPI (blue) staining on paraffin embedded liver sections shown at 600X magnification. (**C**) Western blotting for Perilipin 2.

Liver morphology was similar between the BbsI control and *Ldlr* gRNA groups, with evidence of large lipid droplets often displacing the nucleus (macrovesicular steatosis) as expected from prolonged Western diet feeding (Fig. 6A). Livers from mice receiving the *Ldlr* + *Apob* gRNAs had fewer large lipid droplets, enlarged hepatocytes with centralized nuclei, and evidence of many small lipid droplets, indicative of microvesicular steatosis^26^. To confirm this, we immunostained liver sections for Perilipin 2 (PLIN2), a structural protein present on the surface of lipid droplets (Fig. 6B). Perilipin 2 staining clearly demarcated large lipid droplets in the control and *Ldlr* gRNA groups, while livers from mice treated with *Ldlr* + *Apob* gRNAs showed numerous small lipid droplets per cell with intense staining. These data were further confirmed by Western blotting, where PLIN2 protein levels were dramatically higher with *Apob* disruption, reflecting the increased total surface area of numerous small lipid droplets (Fig. 6C).

To gain insight into the mechanisms underlying the plasma and liver lipid changes, we performed a survey of hepatic genes involved in *de novo* lipogenesis, fatty acid oxidation, cholesterol synthesis, sterol transport, and bile acid metabolism (Supplementary Figure 3). We observed decreased expression of several genes important for fatty acid oxidation (Supplementary Figure 3B) in mice that received the *Ldlr* gRNA or *Ldlr* + *Apob* relative to the control group. While there was suppression of squalene epoxidase (*Sqle*) in the *Ldlr* + *Apob* group, other genes in the cholesterol biosynthetic pathway were not significantly altered (Supplementary Figure 3C). No consistent changes were seen in genes involved in lipogenesis, cholesterol transport, or bile acid metabolism. A recent report using antisense oligonucleotides showed that short term knockdown of *Apob* (three weeks) activated the endoplasmic reticulum stress (ER stress) markers glucose regulated protein 78 (GRP78/BIP) and phosphorylated eukaryotic initiation factor 2 α (p-eIF2α)^27^. Thus we sought to determine if somatic disruption of *Apob* would also result in ER stress. We saw that both groups receiving the *Ldlr* gRNA showed ER stress^28^ as indicated by increases in Bip/Grp-78, Grp94, spliced Xbp1 and Chop relative to the control animals. Interestingly, cleaved Atf6 was increased only in the *Ldlr* gRNA group, and Atf4 was selectively elevated in the *Ldlr* + *Apob* group (Supplementary Figures 4 and 5).

## Discussion

We demonstrate that AAV-CRISPR can be used to efficiently disrupt a metabolic gene (*Ldlr*) in mouse liver, producing systemic phenotypes- hypercholesterolemia and atherosclerosis. We also show that it is possible to simultaneously disrupt a second gene (*Apob*) while maintaining similar levels of *Ldlr* editing. Compensatory disruption of *Apob* dramatically lowered plasma cholesterol, protected from atherosclerosis, and exacerbated hepatic fat accumulation resulting in a microvesicular steatosis. To our knowledge, this is the first report of somatic genome editing for metabolic disease modeling. Our approach is very efficient, producing a pathological phenotype that recapitulates a common human disease. We provide important information about the efficiency and specificity of genome editing with AAV vectors. Most importantly, our approach allows for combinatorial disruption, and can be used for rapid and efficient functional validation of candidate genes.

The reduction in LDLR and Apo B protein levels appeared even greater than the frequency of Indel formation. Several factors may contribute to this disagreement. First, non-parenchymal cells make up approximately 15% of the liver, and these cells are generally not transduced with AAV8 based vectors. Secondly, our analysis excludes insertions or deletions larger than 30 bases, and very large deletions would not have been captured by PCR. Finally, the epigenetic status of the chromatin is also known to play an important role in determining Cas9 accessibility^29^, so it is possible that transcriptionally active alleles are more efficiently edited. For both *Ldlr* and *Apob*, similar results were obtained for mRNA expression before or after the cut site, suggesting that the majority of indels shift the reading frame and promote nonsense mediated decay (NMD) of the mutant transcripts. *Ldlr* disruption was not significantly different between the groups receiving *Ldlr* gRNA and those receiving *Ldlr* + *Apob* gRNAs – an important consideration for studies requiring disruption of multiple genes in parallel. Indels were found at several highly similar predicted BbsI off-target sites in the mouse genome, indicating this common stuffer sequence^24^ encodes a functional gRNA, and therefore should not be considered as a true non-targeting or empty gRNA control. No detectable off-target editing events were identified at the predicted sites for the *Apob* gRNA. We did however observe off-target cutting (~5%) with the *Ldlr* gRNA at a single site in an intron of the *Stx8* gene, which resides 25 kb away from the nearest splice junction.

We identified integration of short ITR sequences within the target site of the *Apob* gene ranging from 10–26% of the total reads. There does not appear to be homology between the ITR’s and this site. ITR’s are known to have recombinogenic properties^30^ and can promote AAV integration into the host genome in the presence of the Rep protein^13^. Since our recombinant vectors do not contain *Rep* or *Cap*, this may reflect the propensity of these hairpin structures to interact with host DNA repair machinery. Integration of AAV vector sequences is a theoretical safety concern, since increased hepatocellular carcinoma incidence has been observed in mice injected with recombinant AAV vectors as neonates^31^,^32^,^33^, although not as adults^34^. A recent study identified numerous integrations of the wild type AAV2 into the human genome in hepatocellular carcinomas, including candidate tumor suppressors^35^. However, analysis of human liver biopsies from patients undergoing gene therapy suggests that recombinant AAV integration events are rare, randomly distributed, and may not pose a major risk of cancer or genotoxicity^36^. Our study appears to be the first report of integration of AAV vector sequences at a cut site generated by CRISPR/Cas9. Site-specific integration events at DSB should now be considered in the development and safety evaluation of AAV-based genome editing vectors for human gene therapy.

Apo B is an essential component of VLDL, IDL, and LDL particles, but studies of its role in liver are challenging since whole body deletion of *Apob* is embryonically lethal. Interestingly, the fatty liver phenotype we observe differs from other studies of liver *Apob* silencing. Previous knockdown of *Apob* with AAV-shRNA and AAV-miRNA resulted in greater than 80% reductions in mRNA levels, and the hepatocytes had large lipid droplets in mice on a chow diet^37^. In the current work, more than 90% knockdown was observed, and these mice exhibited a severe fatty liver with microvesicular steatosis on a Western diet. Our data are in contrast to a recent report that compared knockdown of *Mttp* and *Apob* using antisense oligonucleotides (ASOs), where *Apob* inhibition lowered plasma cholesterol and VLDL secretion but did not result in steatosis^38^. However, our model employs genetic loss-of-function rather than knockdown of mRNA, and is therefore more analogous to patients with hypobetalipoproteinemia (*APOB*)^39^ and abetalipoproteinemia (*MTTP*)^40^. In both cases, defects in hepatic Apo B secretion result in very low levels of ApoB lipoproteins which are often accompanied by hepatic steatosis in addition to intestinal lipid and fat soluble vitamin malabsorption. Liver-directed inhibition of ApoB and/or MTP are being pursued as treatments for severe cases of familial hypercholesterolemia (FH)^41^,^42^. Our studies support the rationale for hepatic Apo B inhibition to lower plasma cholesterol, but also demonstrate severe and potentially dangerous hepatic fat accumulation.

In summary, we introduce liver-directed somatic genome editing as a way to model metabolic disorders. Disruption of *Ldlr* was severe enough to elicit a systemic phenotype- hypercholesterolemia and atherosclerosis, underscoring the importance of the liver in Apo B lipoprotein metabolism. We also achieved a complete protection from hypercholesterolemia and atherosclerosis through compensatory inhibition of hepatic Apo B secretion. Our data shows that highly efficient genome editing is achievable by this approach, but that off-target events may still occur, even with optimal gRNA design. In conclusion, somatic genome editing with AAV-CRISPR is a novel and versatile approach to model metabolic disease, and its use will provide valuable information on the optimal design of genome editing vectors for human gene therapy.

## Materials and Methods

### Plasmid construction

The AAV-gRNA expression vector 1179_pAAV-U6-BbsI-gRNA-CB-EmGFP was generated by cloning a custom U6-gRNA-CB-EmGFP expression cassette into pAAV-TBG-mcs between the SnaBI sites adjacent to each I-terminal repeat (ITR). The E-CRISP algorithm^43^ was used to design gRNA targeting Exon 14 of the murine *Ldlr* and Exon 5 of murine *Apob.* The gRNA sequences were cloned into the BbsI site of plasmid 1179 using the following oligos- Ldlr top 5′-CACCTGCTGCTGGCCAAGGACATG-3′, Ldlr bottom 5′-AAACCATGTCCTTGGCCAGCAGCA-3′, Apob top 5′-CACCTCAAGCTGGCCATTCCTGAA-3′, and Apob bottom 5′-AAACTTCAGGAATGGCCAGCTTGA-3′. The resulting AAV plasmids (1183_pAAV-U6-Ex14-gRNAd-CB-EmGFP and 1184_pAAV-U6-ApoB-gRNA2-CB-EmGFP) both contain a 5 bp insertion in the CB promoter which does not affect EmGFP expression. The complete vector sequences are in Supplemental Methods, and the plasmids will be made publicly available through Addgene.

### AAV production

The Adenoviral helper plasmid pAdDeltaF6 (PL-F-PVADF6) and the AAV8 packaging vector pAAV2/8 (PL-T-PV0007) were obtained from the University of Pennsylvania Vector Core. AAV were packaged in 293T cells using the triple transfection method^44^ and purified by two rounds of CsCl density gradient centrifugation^45^,^46^. The 293T cells were obtained directly from the American Type Tissue Collection (ATCC, CRL-3216) and not further authenticated or tested for mycoplasma contamination. AAV were dialyzed in 10,000 MWCO Slide-A-Lyzer Cassettes (0.5–3.0 mL) against PBS to remove CsCl, and concentrated using an Amicon 100 kDa MWCO centrifugal filtration device (EMD Millipore Cat# UFC910008) prior to storage at −80 °C. AAV were titered by Q-PCR using primers to EmGFP: forward: GCATCGACTTCAAGGAGGAC, reverse: TGCACGCTGCCGTCCTCGATG following DNase digestion as previously described^45^.

### Animals

Cas9 targeted transgenic mice^22^ were obtained from Jackson Laboratories. These mice have been backcrossed to C57BL/6 J, but still exist on a B6/Sv129 mixed background (Gt(ROSA)26Sortm1.1(CAG-Cas9, -EGFP)Fezh/J; Stock #024858). A breeding colony was established and housed with a light cycle from 7 am to 9 pm. Animals were provided free access to food and water and maintained on a standard chow diet before the experiment. Male Cas9-Tg mice were injected with a total AAV dose of 6 × 10^11^ genome copies (GC) (3 × 10^11^ per gRNA) delivered in PBS by intraperitoneal injection at 6.5–9.5 weeks of age. Animals were randomly allocated to the three groups within each cage of littermates at time of injection (BbsI control, *Ldlr* gRNA + BbsI control, *Ldlr* gRNA + *Apob* gRNA). Immediately following AAV injection, animals were switched to a Western type diet containing 21% fat and 0.21% cholesterol (w/w) (Research Diets D12079B). Mice were fasted for 5 hours prior to lipid measurements. Blood was obtained through the retro-orbital plexus using heparinized Natelson collecting tubes (Fisher, 0266810), and plasma was isolated by centrifugation at 10,000 g for 7 minutes at 4 °C. All experiments were approved by the Baylor College of Medicine Institutional Animal Care and Use Committee (IACUC) under protocol number AN-6243, and all procedures were performed in accordance with the relevant guidelines and regulations.

### Quantitative Reverse Transcriptase PCR

RNA was isolated from liver samples using the Qiagen RNeasy Mini Kit (74106). One microgram RNA was reverse transcribed using BioRad iScript (170–8891). The qPCR was completed with 4 μL per reaction of 1:20 diluted cDNA using primers against regions upstream and downstream of the gRNA cut sites for *Ldlr* and *Apob.* Specifically, the *Ldlr* primer sets span exons 10–11 and exons 16–17, and the *Apob* primer sets span exons 2–3, and exons 12–13, respectively. Some primers used in this study have been previously reported^47^,^48^,^49^. All primers are listed in Supplementary Table 1. Data analysis was performed using the ΔΔcT method^50^ and graphed using GraphPad Prism 6.

### Identification of off-target sites

Potential off-target sites in the mouse genome (mm10) were identified and ranked using the recently developed bioinformatics program COSMID https://crispr.bme.gatech.edu/ 23 with the following inclusion criteria, three mismatches without insertions or deletions and two base mismatches with an insertion or deletion (1 bp bulge). The top ranked sites were selected for further investigation by deep sequencing.

### Deep sequencing to quantify CRISPR/Cas9 activity at genomic loci

Genomic DNA was amplified using locus specific primers (Supplementary Table 1) for 35 cycles with an annealing temperature of 62 °C. A second round PCR amplification was performed with an annealing temperature of 65 °C using 2 uL of primary PCR product to add the P5 and P7 adapter sequences and a barcode for each mouse^51^. Barcoded amplicons were purified by gel extraction (Qiagen, 28706). Amplicons for all target regions were pooled in an equimolar ratio and subjected to 2 × 250 paired-end sequencing on the Illumina MiSeq platform. Paired-end reads were filtered by an average Phred quality (*Q*score) greater than 20 and merged into longer single reads from each pair with a minimum overlap of 30 nucleotides using Fast Length Adjustment of Short reads. Alignment to reference sequences and indel quantification was carried out as previously described^52^. Reported indel frequencies and error bounds are mean and S.D. derived from data from 4 animals for each treatment.

### Western Blotting

Livers were homogenized in radioimmunoprecipitation buffer containing 50 mM Tris pH 8.0, 1 mM EDTA, 1% Triton X-100, 0.1% sodium dodecyl sulfate, 0.5% sodium deoxycholate, 150 mM NaCl, and protease inhibitors (Roche, 11836153001) using a Polytron tissue homogenizer (PT2100). Protein concentration was determined using the bicinchinoic acid protein assay (Pierce, 23227). Proteins (50 μg liver lysate or 1 μL plasma) were resolved via SDS-PAGE on 4–12% gradient gels (Life Technologies, NP0322BOX), and then transferred to polyvinylidene fluoride membranes (Millipore, IPFL00010). Membranes were blocked with a 2:1 solution of Odyssey Blocking Buffer (Li-Cor, 927–40000) and PBS plus 0.05% tween-20 (PBS-T) for 1 hour. Western blotting was performed to detect LDLR using a rabbit polyclonal antibody to the C-terminus at a 1:5,000 dilution^53^ and a mouse monoclonal antibody to beta tubulin at 1:1000 dilution (University of Iowa- Developmental Studies Hybridoma Bank, E7). ApoB was detected using a rabbit polyclonal antibody (Meridian Life Sciences, K23300R)^54^. Detection was performed using goat anti-rabbit 680 nm and goat anti-mouse 800 nm secondary antibodies (Rockland, 611-144-002-0.5 and 610-145-002-0.5) with the Odyssey Classic imager (Li-Cor). Western blotting for other proteins was performed in an identical manner, and all antibodies and dilutions are provided in Supplementary Table 3.

### Lipid measurements

Fasted plasma (5 hr) was collected prior to AAV injection, and then at 4, 8, 12, 16 and 20 weeks thereafter. Total cholesterol was measured using the Wako Cholesterol E Reagent (Wako Diagnostics, 439–17501) according to the manufacturer’s instructions. Plasma free fatty acid levels were measured using the Free Fatty Acid Quantitation Kit (Sigma, MAK044–1KT). Gel filtration chromatography was performed on 190 μl of pooled plasma, diluted to 220 μL in TBS (10 mM Tris pH 7.5, 100 mM NaCl, 1 mM EDTA, and 1 mM sodium azide), and loaded onto a 200 μL loop on an Amersham-Pharmacia ÄKTA chromatography system equipped with two Superose HR6 columns in tandem and eluted with TBS at a flow rate of 0.5 mL/min as previously described^55^. Fractions of 1 mL each were collected, and 100 μL of each fraction was assayed for total cholesterol and triglyceride, reported in μg/mL using the Wako Cholesterol E and Thermo Scientific Infinity Triglycerides (TR22421) kits. Livers were homogenized in 3 volumes PBS, diluted and then solubilized 1% deoxycholate, and analyzed with Infinity TG Reagent (Thermo, TR22321) to determine hepatic triglyceride content as previously described^56^. To assess total lipid levels, lipids were extracted from mouse liver using the Bligh-Dyer method^25^, and separated by thin layer chromatography on 20 × 20 cm plates via hexane: diethyl ether: acetic acid (170:30:1). Lipids were resolved with iodine vapor for quantitation and primuline staining for visualization. Staining intensity was quantified using Image Studio Lite (Licor) and normalized to the BbsI control group.

### Atherosclerosis studies

Following 20 weeks of Western diet, mice were sacrificed and aortae were dissected from the aortic root to the ileal bifurcation, and then split longitudinally for *en face* staining with Oil Red O (VWR, AAA12989-14)^57^. Stained aortae were pinned on black paraffin wax using insect pins (Finescience.com, 26002–20) under PBS, and imaged on a Leica M80 dissection microscope, equipped with a Leica IC80 HD camera. Lesion area was manually quantified using the ImageJ^58^ software and expressed as a percentage of total vessel area.

### Histology and immunohistochemistry

Tissues were fixed overnight in 10% formalin and dehydrated in ethanol. Paraffin embedding, sectioning, and hematoxylin and eosin staining were completed by the Texas Digestive Diseases Morphology Core. Slides were imaged at 400× magnification on a Nikon Ci-L brightfield microscope. For immunohistochemistry, five micron sections were deparaffinized and rehydrated using standard methods. Antigen retrieval was completed in sodium citrate buffer (10 mM sodium citrate, 0.05% Tween-20 in PBS, pH 6) in boiling water for 20 minutes followed by three 5-minute PBS rinses. Slides were blocked for an hour in blocking solution (5% goat serum, 3% BSA, 0.3% Triton X100, in phosphate buffered saline) and then incubated with PLIN2 primary antibody at 1:200 overnight. Secondary antibody (Molecular Probes, R3717) was incubated for 2 hours at room temperature and rinsed five times in PBS for 10 minutes. Coverslips were mounted using Dapi Fluormount G (Southern Biotech, 0100–20) and imaged at 600× magnification.

### Statistics

The sample size was determined based upon previous experience with *Ldlr* KO animals, as well as a small pilot study involving animals injected with either saline (*n* = 3) or the *Ldlr* gRNA (*n* = 4) fed a Western diet for 16 weeks. We calculated that *n* = 5 animals per group would provide 80% power to detect differences of 30% in plasma cholesterol with statistical significance set at p < 0.05. The group sizes in the final experiment were as follows: BbsI control (*n* = 5), *Ldlr* gRNA + BbsI control (*n* = 6), *Ldlr* gRNA + *Apob* gRNA (*n* = 6). Animals were randomly allocated to experimental groups within each cage of littermates, and maintained in the same cage throughout the study. Investigators were not blinded during the experiment or when assessing the outcome. Pre-determined exclusion criteria included malocclusion or failed injection based on the absence of detectable AAV genomes. AAV vector genomes were analyzed in all livers by PCR, and one animal in the *Ldlr* gRNA group was excluded for failed injection (final *n* = 5). Data were normally distributed and comparisons were performed using One Way ANOVA followed by Tukey’s posttest where differences exist. All data are biological and not technical replicates, and are presented as the mean or individual data points. In all cases error bars represent the standard deviation within the group, and statistical significance was assigned at *p* < 0.05 (noted with an asterisk*).

## Additional Information

**How to cite this article**: Jarrett, K. E. *et al*. Somatic genome editing with CRISPR/Cas9 generates and corrects a metabolic disease. *Sci. Rep.* **7**, 44624; doi: 10.1038/srep44624 (2017).

**Publisher's note:** Springer Nature remains neutral with regard to jurisdictional claims in published maps and institutional affiliations.

## Supplementary Material

Supplementary Figures

Supplementary Dataset 1

Supplementary Dataset 2

Supplementary Dataset 3
