# Supplementary material for: Somatic genome editing with CRISPR/Cas9 generates and corrects a metabolic disease
Source: Sci Rep. 2017 Mar 16;7:44624. doi: 10.1038/srep44624 (PMC5353616; doi:10.1038/srep44624)
Supplement: Supplementary Figures [file srep44624-s1.pdf]

Supplemental Data and Materials

Somatic genome editing with CRISPR/Cas9 generates and corrects a metabolic disease

Kelsey E. Jarrett[a,b], Ciaran M. Lee[c], Yi-Hsien Yeh[a], Rachel H. Hsu[a], Rajat Gupta[a], Min Zhang[a], Perla J. Rodriguez[b,d], Chang Seok Lee[a], Baiba K. Gillard[d,e], Karl-Dimiter Bissig[f], Henry J. Pownall[d,e], James F. Martin[a,g], Gang Bao[c], William R. Lagor[a,b*]

[a]Department of Molecular Physiology and Biophysics, Baylor College of Medicine, Houston, TX 77030, USA.

[b]Integrative Molecular and Biomedical Sciences Graduate Program, Baylor College of Medicine, Houston, TX 77030, USA

[c]Department of Bioengineering, Rice University, Houston, TX 77030, USA

[d]Houston Methodist Research Institute, Houston, TX 77030, USA

[e]Weill Cornell Medicine, Houston, TX 77030, USA

[f]Center for Cell and Gene Therapy, Department of Molecular and Cellular Biology, Baylor College of Medicine, Houston, Texas 77030, USA

[g]Texas Heart Institute, Houston, TX 77030, USA

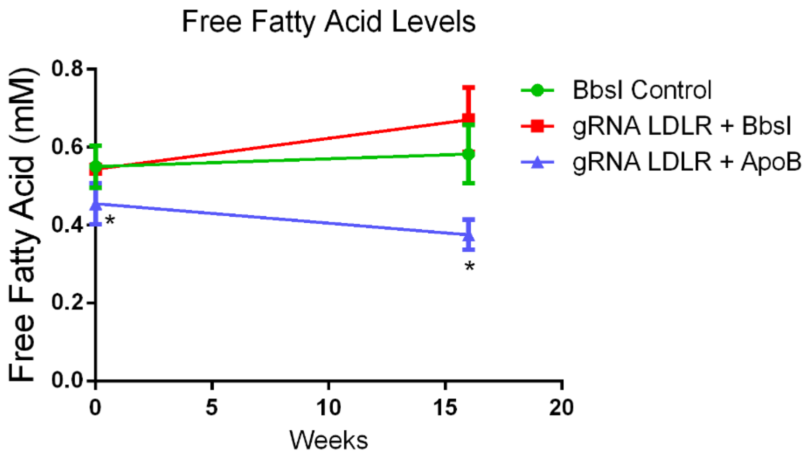

Supplementary Figure 1. Free Fatty Acid Levels in Mouse Plasma. Plasma fatty acids were measured at the start of the experiment and after 16 weeks on western diet. BbsI control *n* = 5, *Ldlr* gRNA + BbsI *n* = 5, *Ldlr* + *Apob* gRNA *n* = 6; data are represented as mean +/- S.D. and *$p<0.05$.

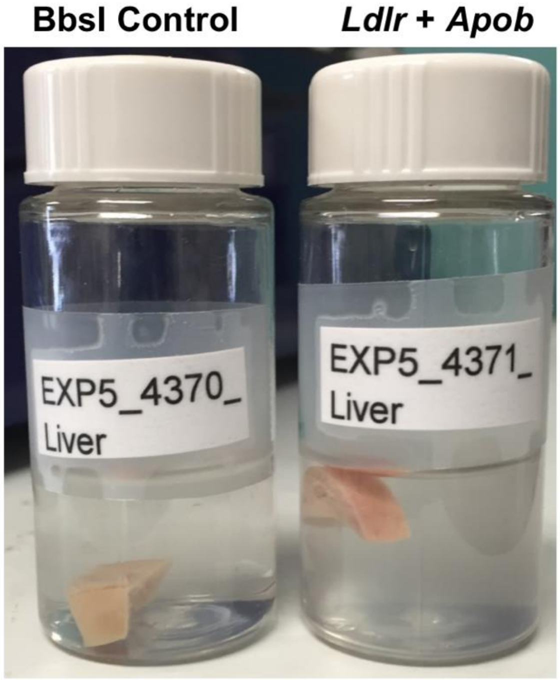

Supplementary Figure 2. Post dissection livers in formalin. Livers from mice in the *Ldlr* + *Apob* group were visibly fatty and were observed to float in formalin. Only livers from this group failed to sink.

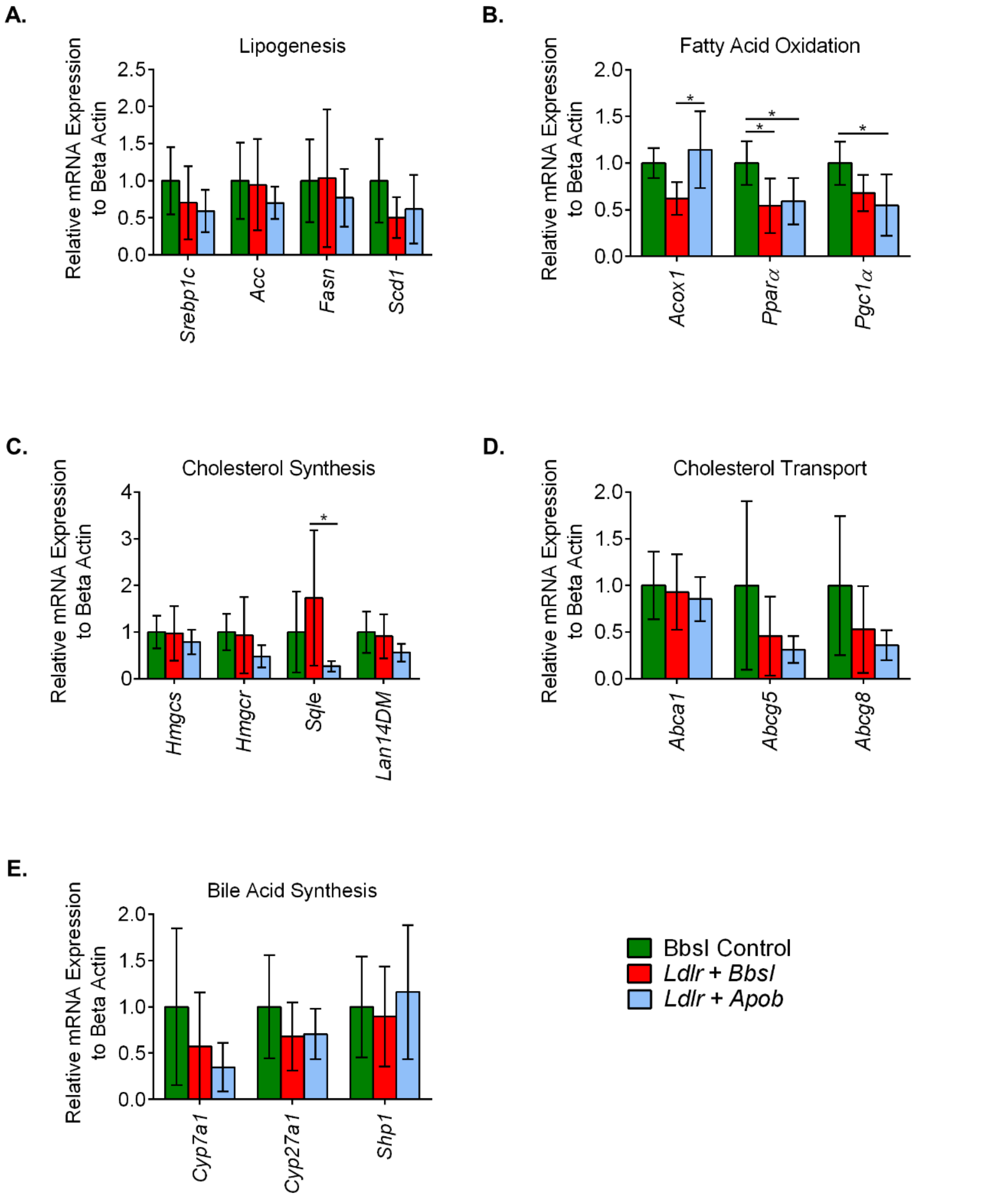

Supplementary Figure 3. Hepatic mRNA expression survey. BbsI control n = 5, *Ldlr* gRNA + BbsI n = 5, *Ldlr* + *Apob* gRNA n = 6; data are represented as mean +/- S.D. and *p<0.05.

A.

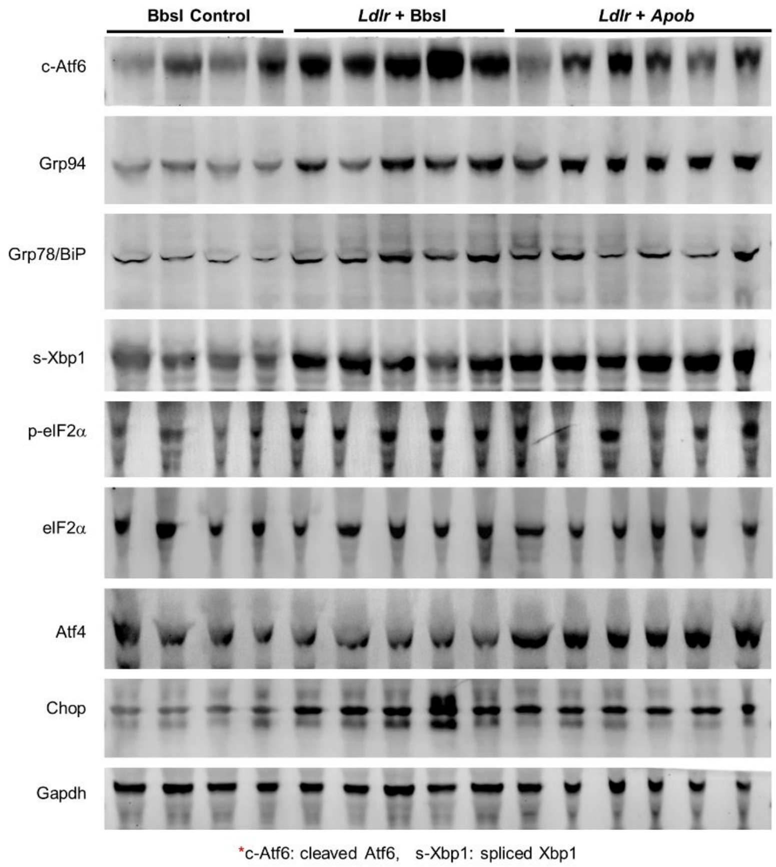

B.

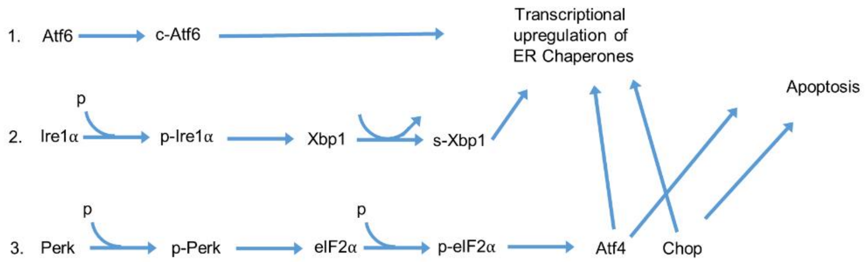

Supplementary Figure 4. Hepatic ER Stress Response Protein Expression. (A) Western blot analysis of ER stress proteins. **(B)** ER Stress pathways modified from Wang and Kaufman, 2014[28]. BbsI control n = 4, *Ldlr* gRNA + BbsI n = 5, *Ldlr* + *Apob* gRNA n = 6; data are represented as mean +/- S.D. and *p<0.05.

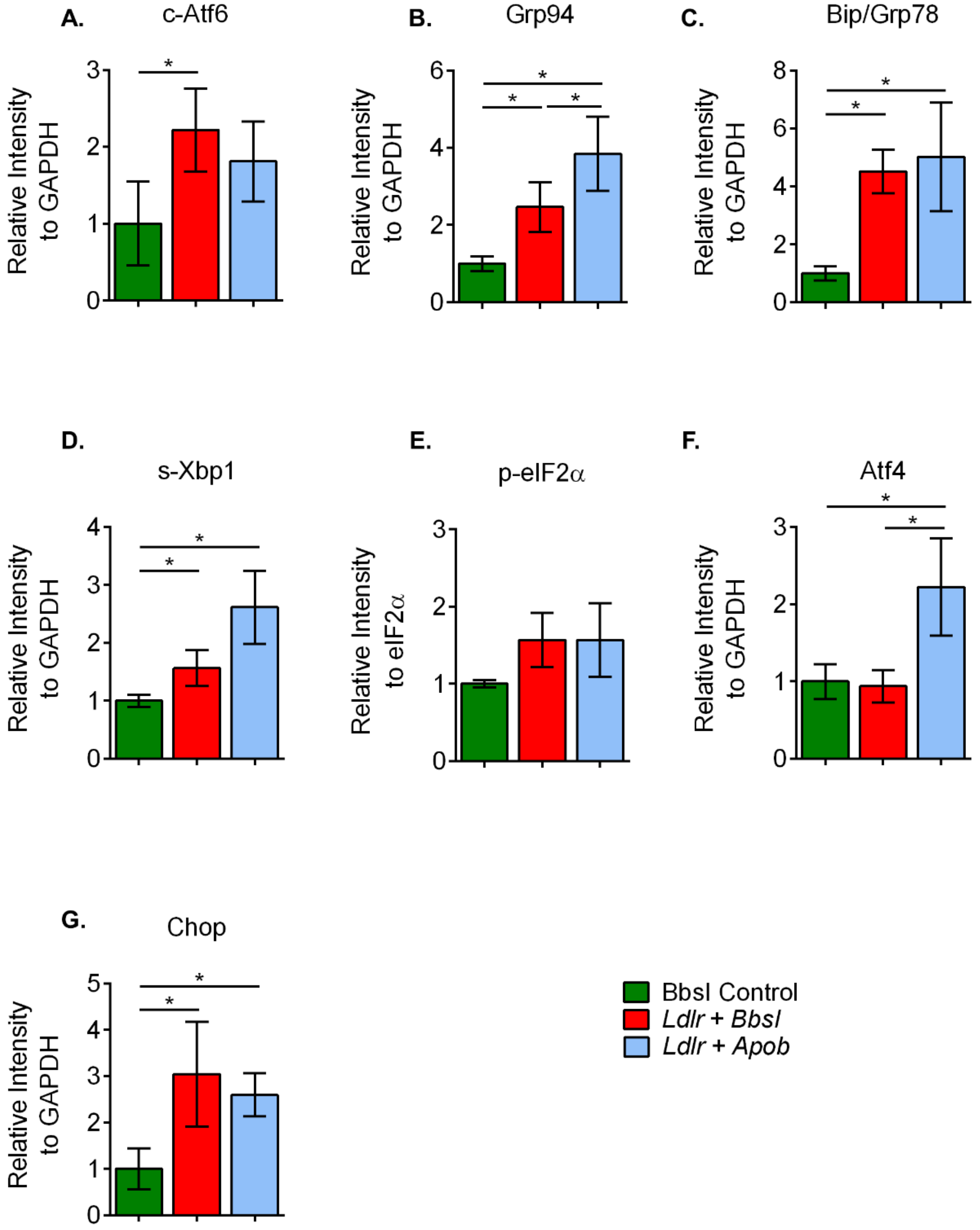

Supplementary Figure 5. Analysis of Hepatic ER Stress Response Protein Expression. Relative intensity of each protein assessed was normalized to GAPDH or eIF2α loading controls. BbsI control n = 4, *Ldlr* gRNA + BbsI n = 5, *Ldlr* + *Apob* gRNA n = 6; data are represented as mean +/- S.D. and *$p<0.05$.

Vector Sequence and Features- The vectors used for AAV gRNA expression are shown below with relevant features and complete sequence. These will be made freely available through Addgene upon publication of the manuscript.

1179_pAAV-U6-BbsI-gRNA-CB-EmGFP

<u>Features</u>

5'ITR (1..185)

U6 promoter (255..495)

CLONING_SITE_FOR_GRNA (504..521)

guideRNA backbone (522..598)

Partial CMV enhancer (681..806)

Chicken Beta Actin promoter (807..1032)

34 bp deletion in Chicken Beta Actin Promoter (following base 903)

Emerald GFP (1083..1802)

Synthetic PolyA (1848..1908)

3'ITR (1909..2081)

pEMBL8 Plasmid Backbone (2106..4521)

>CAGCTGCGCGCTCGCTCGCTCACTGAGGCCGCCCGGGCAAAGCCCGGGCGTCG
GGCGACCTTTGGTCGCCCGGCCTCAGTGAGCGAGCGAGCGCGCAGAGAGGGAGT
GGCCAACTCCATCACTAGGGGTTCCTTGTAGTTAATGATTAACCCGCCATGCTACTT
ATCTACGTAGCCATGCTCTGGTGTACAAAAAAGCAGGCTTTAAAGGAACCAATTCA
GTCGACTGGATCCGGTACCAAGGTCGGGCAGGAAGAGGGCCTATTTCCCATGATT
CCTTCATATTTGCATATACGATACAAGGCTGTTAGAGAGATAATTAGAATTAATTTGA
CTGTAAACACAAAGATATTAGTACAAAATACGTGACGTAGAAAGTAATAATTTCTTG

GGTAGTTTGCAGTTTTAAAATTATGTTTTAAAATGGACTATCATATGCTTACCGTAAC

TTGAAAGTATTTCGATTTCTTGGCTTTATATATCTTGTGGAAAGGACGAAACACCGG

GTCTTCGAGAAGACCTGTTTTAGAGCTAGAAATAGCAAGTTAAAATAAGGCTAGTCC

GTTATCAACTTGAAAAAGTGGCACCGAGTCGGTGCTTTTTTTCTAGACCCAGCTTTC

AAGCTTGATTAACCCGCCATGCTACTTATCTACGTAGCCATGCTCTAGGAAGATCG

GAATTCGCCCTTAAGCTAGTATGCCAAGTACGCCCCCTATTGACGTCAATGACGGT

AAATGGCCCGCCTGGCATTATGCCCAGTACATGACCTTATGGGACTTTCCTACTTG

GCAGTACATCTACTCGAGGCCACGTTCTGCTTCACTCTCCCCATCTCCCCCCCCTC

CCCACCCCCAATTTTGTATTTATTTATTTTTTAATTATTTTGTGCAGCGATGGGGGCG

GGGCGAGGGGCGGGGCGGGGCGAGGCGGAGAGGTGCGGCGGCAGCCAATCAG

AGCGGCGCGCTCCGAAAGTTTCCTTTTATGGCGAGGCGGCGGCGGCGGCGGCCC

TATAAAAAGCGAAGCGCGCGGCGGGCGGGAGCGGGATCAGAATGATCTGATATCA

TCGATGAATTCGAGCTCACCATGGTGAGCAAGGGCGAGGAGCTGTTCACCGGGGT

GGTGCCCATCCTGGTCGAGCTGGACGGCGACGTAAACGGCCACAAGTTCAGCGTG

TCCGGCGAGGGCGAGGGCGATGCCACCTACGGCAAGCTGACCCTGAAGTTCATCT

GCACCACCGGCAAGCTGCCCGTGCCCTGGCCCACCCTCGTGACCACCTTCACCTA

CGGCGTGCAGTGCTTCGCCCGCTACCCCGACCACATGAAGCAGCACGACTTCTTC

AAGTCCGCCATGCCCGAAGGCTACGTCCAGGAGCGCACCATCTTCTTCAAGGACG

ACGGCAACTACAAGACCCGCGCCGAGGTGAAGTTCGAGGGCGACACCCTGGTGAA

CCGCATCGAGCTGAAGGGCATCGACTTCAAGGAGGACGGCAACATCCTGGGGCAC

AAGCTGGAGTACAACTACAACAGCCACAAGGTCTATATCACCGCCGACAAGCAGAA

GAACGGCATCAAGGTGAACTTCAAGACCCGCCACAACATCGAGGACGGCAGCGTG

CAGCTCGCCGACCACTACCAGCAGAACACCCCCATCGGCGACGGCCCCGTGCTG

CTGCCCGACAACCACTACCTGAGCACCCAGTCCGCCCTGAGCAAAGACCCCAACG

AGAAGCGCGATCACATGGTCCTGCTGGAGTTCGTGACCGCCGCCGGGATCACTCT

CGGCATGGACGAGCTGTACAAGTAAGAATTCTGCAGTCGACGCGGGCCCGGGATC

CACCGGTGCGGCCGCTCGCGAATAAAAGATCTTTATTTTCATTAGATCTGTGTGTTG

GTTTTTTGTGTGATGCAGCTACGTAGATAAGTAGCATGGCGGGTTAATCATTAACTA

CAAGGAACCCCTAGTGATGGAGTTGGCCACTCCCTCTCTGCGCGCTCGCTCGCTC

ACTGAGGCCGGGCGACCAAAGGTCGCCCGACGCCCGGGCTTTGCCCGGGCGGCC

TCAGTGAGCGAGCGAGCGCGCAGCTGCATTAATGAATCGGCCAACGCGCGGGGA

GAGGCGGTTTGCGTATTGGGCGCTCTTCCGCTTCCTCGCTCACTGACTCGCTGCG

CTCGGTCGTTCGGCTGCGGCGAGCGGTATCAGCTCACTCAAAGGCGGTAATACGG

TTATCCACAGAATCAGGGGATAACGCAGGAAAGAACATGTGAGCAAAACCGCAGCA

AAAGGCCAGGAACCGTAAAAAGGCCGCGTTGCTGGCGTTTTTCCATAGGCTCCGC

CCCCCTGACGAGCATCACAAAAATCGACGCTCAAGTCAGAGGTGGCGAAACCCGA

CAGGACTATAAAGATACCAGGCGTTTCCCCCTGGAAGCTCCCTCGTGCGCTCTCCT

GTTCCGACCCTGCCGCTTACCGGATACCTGTCCGCCTTTCTCCCTTCGGGAAGCGT

GGCGCTTTCTCATAGCTCACGCTGTAGGTATCTCAGTTCGGTGTAGGTCGTTCGCT

CCAAGCTGGGCTGTGTGCACGAACCCCCCGTTCAGCCCGACCGCTGCGCCTTATC

CGGTAACTATCGTCTTGAGTCCAACCCGGTAAGACACGACTTATCGCCACTGGCAG

CAGCCACTGGTAACAGGATTAGCAGAGCGAGGTATGTAGGCGGTGCTACAGAGTT

CTTGAAGTGGTGGCCTAACTACGGCTACACTAGAAGGACAGTATTTGGTATCTGCG

CTCTGCTGAAGCCAGTTACCTTCGGAAAAAGAGTTGGTAGCTCTTGATCCGGCAAA

CAAACCACCGCTGGTAGCGGTGGTTTTTTTGTTTGCAAGCAGCAGATTACGCGCAG

AAAAAAAGGATCTCAAGAAGATCCTTTGATCTTTTCTACGGGGTCTGACGCTCAGTG

GAACGAAAACTCACGTTAAGGGATTTTGGTCATGAGATTATCAAAAAGGATCTTCAC

CTAGATCCTTTTAAATTAAAAATGAAGTTTTAAATCAATCTAAAGTATATATGAGTAAA

CTTGGTCTGACAGTTACCAATGCTTAATCAGTGAGGCACCTATCTCAGCGATCTGTC

TATTTCGTTCATCCATAGTTGCCTGACTCCCCGTCGTGTAGATAACTACGATACGGG

AGGGCTTACCATCTGGCCCCAGTGCTGCAATGATACCGCGAGACCCACGCTCACC

GGCTCCAGATTTATCAGCAATAAACCAGCCAGCCGGAAGGGCCGAGCGCAGAAGT

GGTCCTGCAACTTTATCCGCCTCCATCCAGTCTATTAATTGTTGCCGGGAAGCTAG

AGTAAGTAGTTCGCCAGTTAATAGTTTGCGCAACGTTGTTACCATTACTACAGGCAT

CGTGGTGTCACGCTCGTCGTTTGGTATGGCTTCATTCAGCTCCGGTTCCCAACGAT

CAAGGCGAGTTACATGATCCCCCATGTTGTGCAAAAAAGCGGTTAGCTCCTTCGGT

CCTCCGATCGTTGTCAGAAGTAAGTTGGCCGCAGTGTTATCACTCATGGTTATGGC

AGCACTGCATAATTCTCTTACTGTCATGCCATCCGTAAGATGCTTTTCTGTGACTGG

TGAGTACTCAACCAAGTCATTCTGAGAATAGTGTATGCGGCGACCGAGTTGCTCTT

GCCCGGCGTCAATACGGGATAATACCGCGCCACATAGCAGAACTTTAAAAGTGCTC

ATCATTGGAAAACGTTCTTCGGGGCGAAAACTCTCAAGGATCTTACCACTATTGAGA

TCCAGTTCGATGTAACCCACTCGTGCACCCAACTGATCTTCAGCATCTTTTACTTTC

ACCAGCGTTTCTGGGTGAGCAAAAACAGGAAGGCAAAATGCCGCAAAAAAGGGAA

TAAGGGCGACACGGAAATGTTGAATACTCATACTCTTCCTTTTTCAATATTATTGAAG

CATTTATCAGGGTTATTGTCTCATGAGCGGATACATATTTGAATGTATTTAGAAAAAT

AAACAAATAGGGGTTCCGCGCACATTTCCCCGAAAAGATGCCACCTGAAATTATAAA

CGTTAATATTTTGTTAAAATTCGCGTTAAATTTTTGTTAAATCAGCTCATTTTTTAACC

AATAGGCCGAAATCGGAAAAATCCCTTATAAATCAAAAGAATAGACCGAGATAGGG

TTGAGTGTTGTTCCAGTTTGGAACAAGAGTCCACTATTGAGGAACGTGAACTCCAG

CGTCAAAGGGCGAAAAACCGTCTATCGGGGCGATGGCCCACTACGTGAACCATCA

CCCTAATCAAGTTTTTTGGGGTCGAGGTGCCGTAAAGCACTAAATCGGAACCCTAA

AGGGAGCCCCCGATTTAGAGCTTGACGGGGAAAGCCGGCGAACGTGGCGAGAAA

GGAAGGGAAGAAAGCGAAAGGAGCGGGCGCTAGGGCGCTGGCAAGTGTAGCGGT

CACGCTGCGCGTAACCACCACACCCGCCGCGCTTAATGCGCCGCTACAGGGCGC

GTCCCATTCGCCATTCAGGCTGCGCAACTGTTGGGAAGGGCGATCGGTGCGGGCC

TCTTCGCTATTACGC

1183_pAAV-U6-Ex14-gRNAd-CB-EmGFP

Features

5'ITR (1..185)

U6 promoter (255..495)

Ex14gRNA-d (504..523)

guideRNA backbone (524..600)

Partial CMV enhancer (683..808)

Chicken Beta Actin promoter (809..1039)

Deletion of 34 bp in Chicken Beta Actin Promoter (following base 903)

Insertion of 5 bp in Chicken Beta Actin Promoter (909..913)

Emerald GFP (1090..1809)

Synthetic PolyA (1855..1915)

3'ITR (1916..2088)

pEMBL8 Plasmid Backbone (2113..4528)

>CAGCTGCGCGCTCGCTCGCTCACTGAGGCCGCCCGGGCAAAGCCCGGGCGTCG

GGCGACCTTTGGTCGCCCGGCCTCAGTGAGCGAGCGAGCGCGCAGAGAGGGAGT

GGCCAACTCCATCACTAGGGGTTCCTTGTAGTTAATGATTAACCCGCCATGCTACTT

ATCTACGTAGCCATGCTCTGGTGTACAAAAAAGCAGGCTTTAAAGGAACCAATTCA

GTCGACTGGATCCGGTACCAAGGTCGGGCAGGAAGAGGGCCTATTTCCCATGATT

CCTTCATATTTGCATATACGATACAAGGCTGTTAGAGAGATAATTAGAATTAATTTGA

CTGTAAACACAAAGATATTAGTACAAAATACGTGACGTAGAAAGTAATAATTTCTTG

GGTAGTTTGCAGTTTTAAAATTATGTTTTAAAATGGACTATCATATGCTTACCGTAAC

TTGAAAGTATTTCGATTTCTTGGCTTTATATATCTTGTGGAAAGGACGAAACACCTG
CTGCTGGCCAAGGACATGGTTTTAGAGCTAGAAATAGCAAGTTAAAATAAGGCTAG
TCCGTTATCAACTTGAAAAAGTGGCACCGAGTCGGTGCTTTTTTTCTAGACCCAGCT
TTCAAGCTTGATTAACCCGCCATGCTACTTATCTACGTAGCCATGCTCTAGGAAGAT
CGGAATTCGCCCTTAAGCTAGTATGCCAAGTACGCCCCCTATTGACGTCAATGACG
GTAAATGGCCCGCCTGGCATTATGCCCAGTACATGACCTTATGGGACTTTCCTACT
TGGCAGTACATCTACTCGAGGCCACGTTCTGCTTCACTCTCCCCATCTCCCCCCCC
TCCCCACCCCCAATTTTGTATTTATTTATTTTTTAATTATTTTGTGCAGCGATGGGGG
CGGGGCGGGGCGAGGGGCGGGGCGGGGCGAGGCGGAGAGGTGCGGCGGCAGC
CAATCAGAGCGGCGCGCTCCGAAAGTTTCCTTTTATGGCGAGGCGGCGGCGGCG
GCGGCCCTATAAAAAGCGAAGCGCGCGGCGGGCGGGAGCGGGATCAGAATGATC
TGATATCATCGATGAATTCGAGCTCACCATGGTGAGCAAGGGCGAGGAGCTGTTCA
CCGGGGTGGTGCCCATCCTGGTCGAGCTGGACGGCGACGTAAACGGCCACAAGT
TCAGCGTGTCCGGCGAGGGCGAGGGCGATGCCACCTACGGCAAGCTGACCCTGA
AGTTCATCTGCACCACCGGCAAGCTGCCCGTGCCCTGGCCCACCCTCGTGACCAC
CTTCACCTACGGCGTGCAGTGCTTCGCCCGCTACCCCGACCACATGAAGCAGCAC
GACTTCTTCAAGTCCGCCATGCCCGAAGGCTACGTCCAGGAGCGCACCATCTTCTT
CAAGGACGACGGCAACTACAAGACCCGCGCCGAGGTGAAGTTCGAGGGCGACAC
CCTGGTGAACCGCATCGAGCTGAAGGGCATCGACTTCAAGGAGGACGGCAACATC
CTGGGGCACAAGCTGGAGTACAACTACAACAGCCACAAGGTCTATATCACCGCCG
ACAAGCAGAAGAACGGCATCAAGGTGAACTTCAAGACCCGCCACAACATCGAGGA
CGGCAGCGTGCAGCTCGCCGACCACTACCAGCAGAACACCCCCATCGGCGACGG
CCCCGTGCTGCTGCCCGACAACCACTACCTGAGCACCCAGTCCGCCCTGAGCAAA
GACCCCAACGAGAAGCGCGATCACATGGTCCTGCTGGAGTTCGTGACCGCCGCCG
GGATCACTCTCGGCATGGACGAGCTGTACAAGTAAGAATTCTGCAGTCGACGCGG
GCCCGGGATCCACCGGTGCGGCCGCTCGCGAATAAAAGATCTTTATTTTCATTAGA

TCTGTGTGTTGGTTTTTTGTGTGATGCAGCTACGTAGATAAGTAGCATGGCGGGTTA

ATCATTAACTACAAGGAACCCCTAGTGATGGAGTTGGCCACTCCCTCTCTGCGCGC

TCGCTCGCTCACTGAGGCCGGGCGACCAAAGGTCGCCCGACGCCCGGGCTTTGC

CCGGGCGGCCTCAGTGAGCGAGCGAGCGCGCAGCTGCATTAATGAATCGGCCAA

CGCGCGGGGAGAGGCGGTTTGCGTATTGGGCGCTCTTCCGCTTCCTCGCTCACTG

ACTCGCTGCGCTCGGTCGTTCGGCTGCGGCGAGCGGTATCAGCTCACTCAAAGGC

GGTAATACGGTTATCCACAGAATCAGGGGATAACGCAGGAAAGAACATGTGAGCAA

AACCGCAGCAAAAGGCCAGGAACCGTAAAAAGGCCGCGTTGCTGGCGTTTTTCCA

TAGGCTCCGCCCCCCTGACGAGCATCACAAAAATCGACGCTCAAGTCAGAGGTGG

CGAAACCCGACAGGACTATAAAGATACCAGGCGTTTCCCCCTGGAAGCTCCCTCGT

GCGCTCTCCTGTTCCGACCCTGCCGCTTACCGGATACCTGTCCGCCTTTCTCCCTT

CGGGAAGCGTGGCGCTTTCTCATAGCTCACGCTGTAGGTATCTCAGTTCGGTGTAG

GTCGTTCGCTCCAAGCTGGGCTGTGTGCACGAACCCCCCGTTCAGCCCGACCGCT

GCGCCTTATCCGGTAACTATCGTCTTGAGTCCAACCCGGTAAGACACGACTTATCG

CCACTGGCAGCAGCCACTGGTAACAGGATTAGCAGAGCGAGGTATGTAGGCGGTG

CTACAGAGTTCTTGAAGTGGTGGCCTAACTACGGCTACACTAGAAGGACAGTATTT

GGTATCTGCGCTCTGCTGAAGCCAGTTACCTTCGGAAAAAGAGTTGGTAGCTCTTG

ATCCGGCAAACAAACCACCGCTGGTAGCGGTGGTTTTTTTGTTTGCAAGCAGCAGA

TTACGCGCAGAAAAAAAGGATCTCAAGAAGATCCTTTGATCTTTTCTACGGGGTCTG

ACGCTCAGTGGAACGAAAACTCACGTTAAGGGATTTTGGTCATGAGATTATCAAAAA

GGATCTTCACCTAGATCCTTTTAAATTAAAAATGAAGTTTTAAATCAATCTAAAGTAT

ATATGAGTAAACTTGGTCTGACAGTTACCAATGCTTAATCAGTGAGGCACCTATCTC

AGCGATCTGTCTATTTCGTTCATCCATAGTTGCCTGACTCCCCGTCGTGTAGATAAC

TACGATACGGGAGGGCTTACCATCTGGCCCCAGTGCTGCAATGATACCGCGAGAC

CCACGCTCACCGGCTCCAGATTTATCAGCAATAAACCAGCCAGCCGGAAGGGCCG

AGCGCAGAAGTGGTCCTGCAACTTTATCCGCCTCCATCCAGTCTATTAATTGTTGCC

GGGAAGCTAGAGTAAGTAGTTCGCCAGTTAATAGTTTGCGCAACGTTGTTACCATTA
CTACAGGCATCGTGGTGTCACGCTCGTCGTTTGGTATGGCTTCATTCAGCTCCGGT
TCCCAACGATCAAGGCGAGTTACATGATCCCCCATGTTGTGCAAAAAAGCGGTTAG
CTCCTTCGGTCCTCCGATCGTTGTCAGAAGTAAGTTGGCCGCAGTGTTATCACTCA
TGGTTATGGCAGCACTGCATAATTCTCTTACTGTCATGCCATCCGTAAGATGCTTTT
CTGTGACTGGTGAGTACTCAACCAAGTCATTCTGAGAATAGTGTATGCGGCGACCG
AGTTGCTCTTGCCCGGCGTCAATACGGGATAATACCGCGCCACATAGCAGAACTTT
AAAAGTGCTCATCATTGGAAAACGTTCTTCGGGGCGAAAACTCTCAAGGATCTTAC
CACTATTGAGATCCAGTTCGATGTAACCCACTCGTGCACCCAACTGATCTTCAGCAT
CTTTTACTTTCACCAGCGTTTCTGGGTGAGCAAAAACAGGAAGGCAAAATGCCGCA
AAAAAGGGAATAAGGGCGACACGGAAATGTTGAATACTCATACTCTTCCTTTTTCAA
TATTATTGAAGCATTTATCAGGGTTATTGTCTCATGAGCGGATACATATTTGAATGTA
TTTAGAAAAATAAACAAATAGGGGTTCCGCGCACATTTCCCCGAAAGATGCCACCT
GAAATTATAAACGTTAATATTTTGTTAAAATTCGCGTTAAATTTTTGTTAAATCAGCTC
ATTTTTTAACCAATAGGCCGAAATCGGAAAAATCCCTTATAAATCAAAAGAATAGAC
CGAGATAGGGTTGAGTGTTGTTCCAGTTTGGAACAAGAGTCCACTATTGAGGAACG
TGAACTCCAGCGTCAAAGGGCGAAAAACCGTCTATCGGGGCGATGGCCCACTACG
TGAACCATCACCCTAATCAAGTTTTTTGGGGTCGAGGTGCCGTAAAGCACTAAATC
GGAACCCTAAAGGGAGCCCCCGATTTAGAGCTTGACGGGGAAAGCCGGCGAACGT
GGCGAGAAAGGAAGGGAAGAAAGCGAAAGGAGCGGGCGCTAGGGCGCTGGCAAG
TGTAGCGGTCACGCTGCGCGTAACCACCACACCCGCCGCGCTTAATGCGCCGCTA
CAGGGCGCGTCCCATTCGCCATTCAGGCTGCGCAACTGTTGGGAAGGGCGATCGG
TGCGGGCCTCTTCGCTATTACGC

1184_pAAV-U6-ApoB-gRNA2-CB-EmGFP

<u>Features</u>

5'ITR (1..185)

U6 promoter (255..495)

Ex14gRNA-d (504..523)

guideRNA backbone (524..600)

Partial CMV enhancer (683..808)

Chicken Beta Actin promoter (809..1039)

Deletion of 34 bp in Chicken Beta Actin Promoter (following base 903)

Insertion of 5 bp in Chicken Beta Actin Promoter (909..913)

Emerald GFP (1090..1809)

Synthetic PolyA (1855..1915)

3'ITR (1916..2088)

pEMBL8 Plasmid Backbone (2113..4528)

CAGCTGCGCGCTCGCTCGCTCACTGAGGCCGCCCGGGCAAAGCCCGGGCGTCGG

GCGACCTTTGGTCGCCCGGCCTCAGTGAGCGAGCGAGCGCGCAGAGAGGGAGTG

GCCAACTCCATCACTAGGGGTTCCTTGTAGTTAATGATTAACCCGCCATGCTACTTA

TCTACGTAGCCATGCTCTGGTGTACAAAAAAGCAGGCTTTAAAGGAACCAATTCAGT

CGACTGGATCCGGTACCAAGGTCGGGCAGGAAGAGGGCCTATTTCCCATGATTCC

TTCATATTTGCATATACGATACAAGGCTGTTAGAGAGATAATTAGAATTAATTTGACT

GTAAACACAAAGATATTAGTACAAAATACGTGACGTAGAAAGTAATAATTTCTTGGG

TAGTTTGCAGTTTTAAAATTATGTTTTAAAATGGACTATCATATGCTTACCGTAACTT

GAAAGTATTTCGATTTCTTGGCTTTATATATCTTGTGGAAAGGACGAAACACCTCAA

GCTGGCCATTCCTGAAGTTTTAGAGCTAGAAATAGCAAGTTAAAATAAGGCTAGTCC

GTTATCAACTTGAAAAAGTGGCACCGAGTCGGTGCTTTTTTTCTAGACCCAGCTTTC

AAGCTTGATTAACCCGCCATGCTACTTATCTACGTAGCCATGCTCTAGGAAGATCG

GAATTCGCCCTTAAGCTAGTATGCCAAGTACGCCCCCTATTGACGTCAATGACGGT

AAATGGCCCGCCTGGCATTATGCCCAGTACATGACCTTATGGGACTTTCCTACTTG

GCAGTACATCTACTCGAGGCCACGTTCTGCTTCACTCTCCCCATCTCCCCCCCCTC

CCCACCCCCAATTTTGTATTTATTTATTTTTTAATTATTTTGTGCAGCGATGGGGGCG

GGGCGGGGCGAGGGGCGGGGCGGGGCGAGGCGGAGAGGTGCGGCGGCAGCCA

ATCAGAGCGGCGCGCTCCGAAAGTTTCCTTTTATGGCGAGGCGGCGGCGGCGGC

GGCCCTATAAAAAGCGAAGCGCGCGGCGGGCGGGAGCGGGATCAGAATGATCTG

ATATCATCGATGAATTCGAGCTCACCATGGTGAGCAAGGGCGAGGAGCTGTTCACC

GGGGTGGTGCCCATCCTGGTCGAGCTGGACGGCGACGTAAACGGCCACAAGTTCA

GCGTGTCCGGCGAGGGCGAGGGCGATGCCACCTACGGCAAGCTGACCCTGAAGT

TCATCTGCACCACCGGCAAGCTGCCCGTGCCCTGGCCCACCCTCGTGACCACCTT

CACCTACGGCGTGCAGTGCTTCGCCCGCTACCCCGACCACATGAAGCAGCACGAC

TTCTTCAAGTCCGCCATGCCCGAAGGCTACGTCCAGGAGCGCACCATCTTCTTCAA

GGACGACGGCAACTACAAGACCCGCGCCGAGGTGAAGTTCGAGGGCGACACCCT

GGTGAACCGCATCGAGCTGAAGGGCATCGACTTCAAGGAGGACGGCAACATCCTG

GGGCACAAGCTGGAGTACAACTACAACAGCCACAAGGTCTATATCACCGCCGACAA

GCAGAAGAACGGCATCAAGGTGAACTTCAAGACCCGCCACAACATCGAGGACGGC

AGCGTGCAGCTCGCCGACCACTACCAGCAGAACACCCCCATCGGCGACGGCCCC

GTGCTGCTGCCCGACAACCACTACCTGAGCACCCAGTCCGCCCTGAGCAAAGACC

CCAACGAGAAGCGCGATCACATGGTCCTGCTGGAGTTCGTGACCGCCGCCGGGAT

CACTCTCGGCATGGACGAGCTGTACAAGTAAGAATTCTGCAGTCGACGCGGGCCC

GGGATCCACCGGTGCGGCCGCTCGCGAATAAAAGATCTTTATTTTCATTAGATCTG

TGTGTTGGTTTTTTGTGTGATGCAGCTACGTAGATAAGTAGCATGGCGGGTTAATCA

TTAACTACAAGGAACCCCTAGTGATGGAGTTGGCCACTCCCTCTCTGCGCGCTCGC

TCGCTCACTGAGGCCGGGCGACCAAAGGTCGCCCGACGCCCGGGCTTTGCCCGG
GCGGCCTCAGTGAGCGAGCGAGCGCGCAGCTGCATTAATGAATCGGCCAACGCG
CGGGGAGAGGCGGTTTGCGTATTGGGCGCTCTTCCGCTTCCTCGCTCACTGACTC
GCTGCGCTCGGTCGTTCGGCTGCGGCGAGCGGTATCAGCTCACTCAAAGGCGGTA
ATACGGTTATCCACAGAATCAGGGGATAACGCAGGAAAGAACATGTGAGCAAAACC
GCAGCAAAAGGCCAGGAACCGTAAAAAGGCCGCGTTGCTGGCGTTTTTCCATAGG
CTCCGCCCCCCTGACGAGCATCACAAAAATCGACGCTCAAGTCAGAGGTGGCGAA
ACCCGACAGGACTATAAAGATACCAGGCGTTTCCCCCTGGAAGCTCCCTCGTGCG
CTCTCCTGTTCCGACCCTGCCGCTTACCGGATACCTGTCCGCCTTTCTCCCTTCGG
GAAGCGTGGCGCTTTCTCATAGCTCACGCTGTAGGTATCTCAGTTCGGTGTAGGTC
GTTCGCTCCAAGCTGGGCTGTGTGCACGAACCCCCCGTTCAGCCCGACCGCTGCG
CCTTATCCGGTAACTATCGTCTTGAGTCCAACCCGGTAAGACACGACTTATCGCCA
CTGGCAGCAGCCACTGGTAACAGGATTAGCAGAGCGAGGTATGTAGGCGGTGCTA
CAGAGTTCTTGAAGTGGTGGCCTAACTACGGCTACACTAGAAGGACAGTATTTGGT
ATCTGCGCTCTGCTGAAGCCAGTTACCTTCGGAAAAAGAGTTGGTAGCTCTTGATC
CGGCAAACAAACCACCGCTGGTAGCGGTGGTTTTTTTGTTTGCAAGCAGCAGATTA
CGCGCAGAAAAAAAGGATCTCAAGAAGATCCTTTGATCTTTTCTACGGGGTCTGAC
GCTCAGTGGAACGAAAACTCACGTTAAGGGATTTTGGTCATGAGATTATCAAAAAG
GATCTTCACCTAGATCCTTTTAAATTAAAAATGAAGTTTTAAATCAATCTAAAGTATAT
ATGAGTAAACTTGGTCTGACAGTTACCAATGCTTAATCAGTGAGGCACCTATCTCAG
CGATCTGTCTATTTCGTTCATCCATAGTTGCCTGACTCCCCGTCGTGTAGATAACTA
CGATACGGGAGGGCTTACCATCTGGCCCCAGTGCTGCAATGATACCGCGAGACCC
ACGCTCACCGGCTCCAGATTTATCAGCAATAAACCAGCCAGCCGGAAGGGCCGAG
CGCAGAAGTGGTCCTGCAACTTTATCCGCCTCCATCCAGTCTATTAATTGTTGCCG
GGAAGCTAGAGTAAGTAGTTCGCCAGTTAATAGTTTGCGCAACGTTGTTACCATTAC
TACAGGCATCGTGGTGTCACGCTCGTCGTTTGGTATGGCTTCATTCAGCTCCGGTT

CCCAACGATCAAGGCGAGTTACATGATCCCCCATGTTGTGCAAAAAAGCGGTTAGC
TCCTTCGGTCCTCCGATCGTTGTCAGAAGTAAGTTGGCCGCAGTGTTATCACTCAT
GGTTATGGCAGCACTGCATAATTCTCTTACTGTCATGCCATCCGTAAGATGCTTTTC
TGTGACTGGTGAGTACTCAACCAAGTCATTCTGAGAATAGTGTATGCGGCGACCGA
GTTGCTCTTGCCCGGCGTCAATACGGGATAATACCGCGCCACATAGCAGAACTTTA
AAAGTGCTCATCATTGGAAAACGTTCTTCGGGGCGAAAACTCTCAAGGATCTTACC
ACTATTGAGATCCAGTTCGATGTAACCCACTCGTGCACCCAACTGATCTTCAGCATC
TTTTACTTTCACCAGCGTTTCTGGGTGAGCAAAAACAGGAAGGCAAAATGCCGCAA
AAAAGGGAATAAGGGCGACACGGAAATGTTGAATACTCATACTCTTCCTTTTTCAAT
ATTATTGAAGCATTTATCAGGGTTATTGTCTCATGAGCGGATACATATTTGAATGTAT
TTAGAAAAATAAACAAATAGGGGTTCCGCGCACATTTCCCCGAAAGATGCCACCTG
AAATTATAAACGTTAATATTTTGTTAAAATTCGCGTTAAATTTTTGTTAAATCAGCTCA
TTTTTTAACCAATAGGCCGAAATCGGAAAAATCCCTTATAAATCAAAAGAATAGACC
GAGATAGGGTTGAGTGTTGTTCCAGTTTGGAACAAGAGTCCACTATTGAGGAACGT
GAACTCCAGCGTCAAAGGGCGAAAAACCGTCTATCGGGGCGATGGCCCACTACGT
GAACCATCACCCTAATCAAGTTTTTTGGGGTCGAGGTGCCGTAAAGCACTAAATCG
GAACCCTAAAGGGAGCCCCCGATTTAGAGCTTGACGGGGAAAGCCGGCGAACGTG
GCGAGAAAGGAAGGGAAGAAAGCGAAAGGAGCGGGCGCTAGGGCGCTGGCAAGT
GTAGCGGTCACGCTGCGCGTAACCACCACACCCGCCGCGCTTAATGCGCCGCTAC
AGGGCGCGTCCCATTCGCCATTCAGGCTGCGCAACTGTTGGGAAGGGCGATCGGT
GCGGGCCTCTTCGCTATTACGC

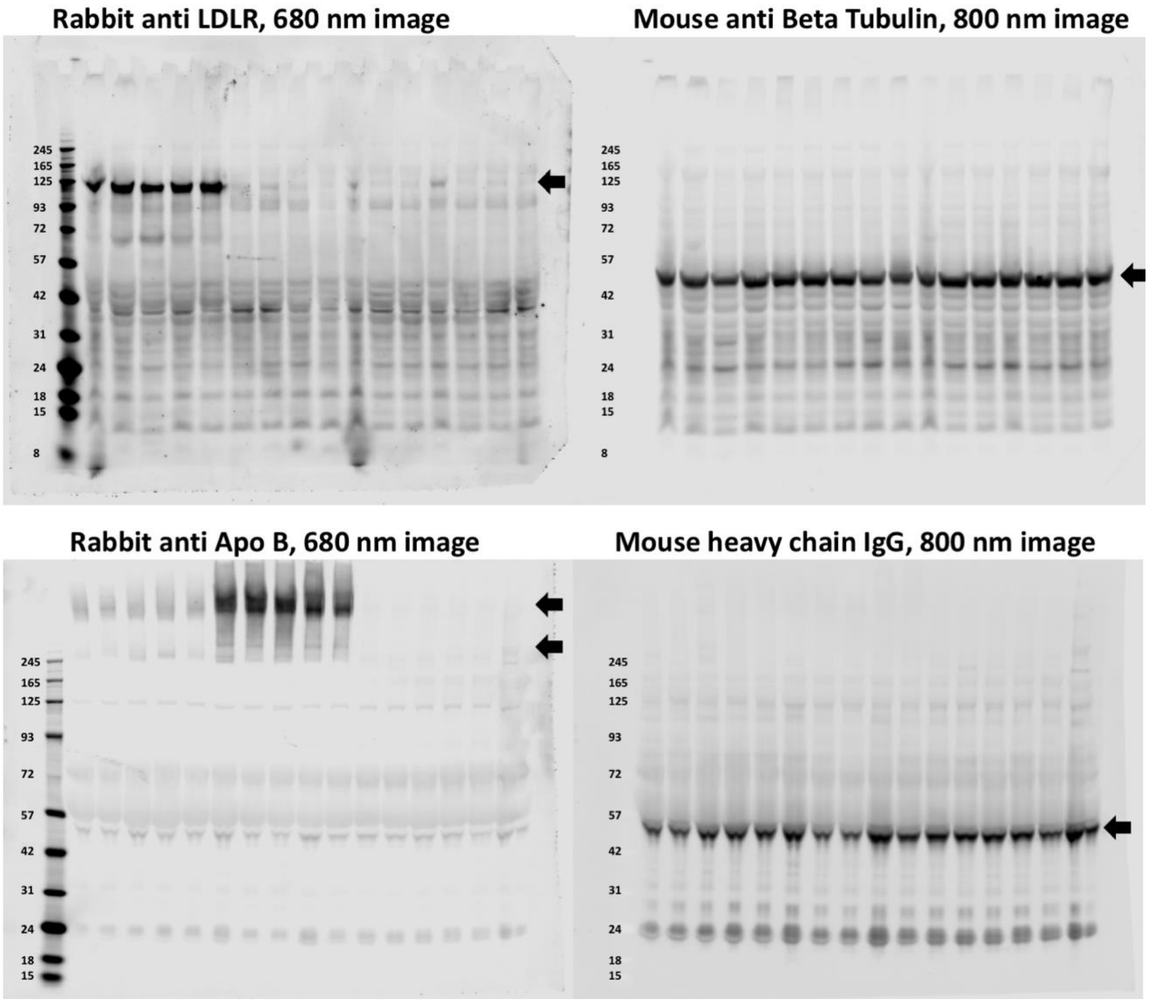

Raw Western Blot Images from Figure 1. These raw western blot images were shown in cropped form in Figure 1C and 1D. LDLR and the beta tubulin control were blotted on the same membrane. Apo B and heavy chain IgG were blotted on the same membrane.

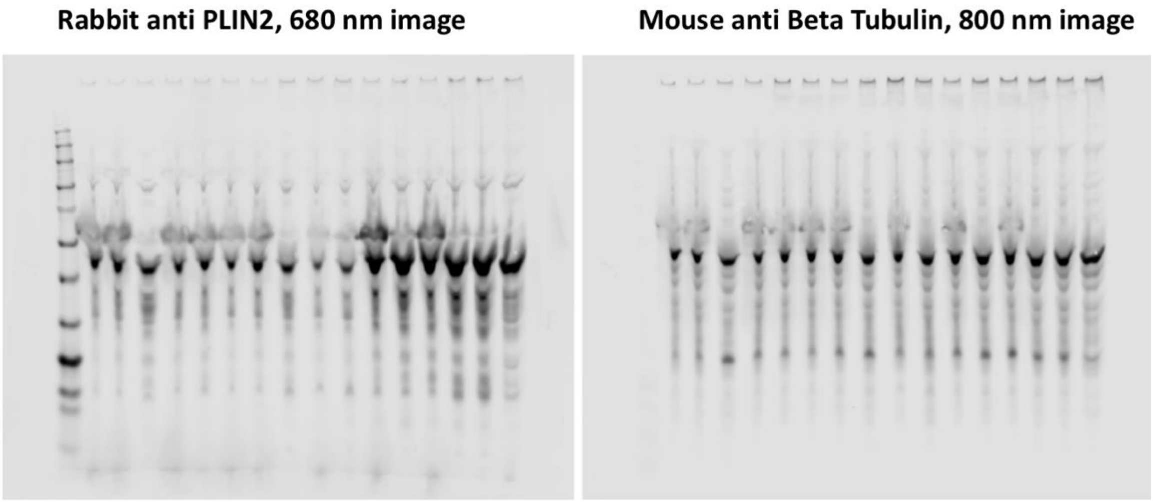

Raw Western Blot Images from Figure 6. These raw western blot images were shown in cropped form in Figure 6c. PLIN2 and the beta tubulin control were blotted on the same membrane.

Mouse anti Atf6, 680 nm image

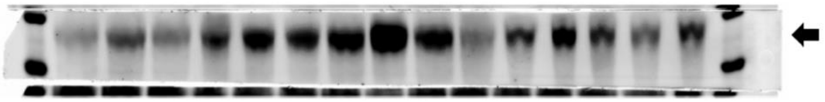

Rabbit anti Grp94, 680 nm image

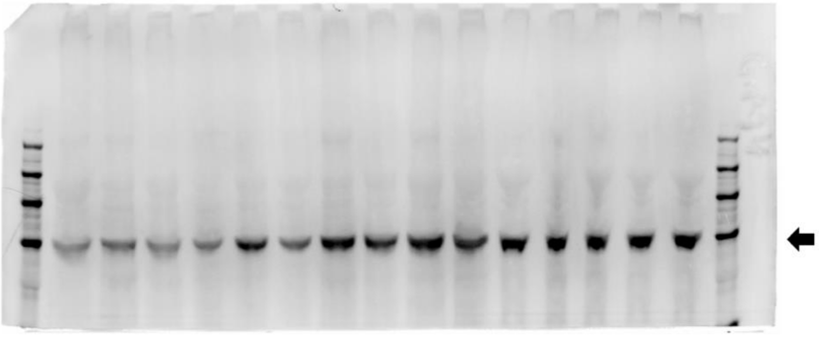

Rabbit anti Bip/Grp78, 680 nm image

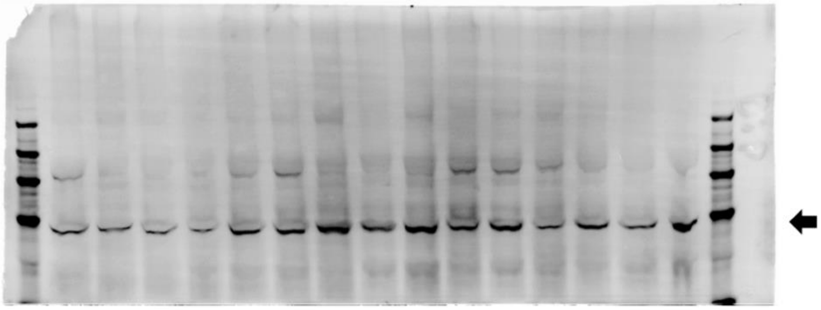

Rabbit anti s-Xbp1, 680 nm image

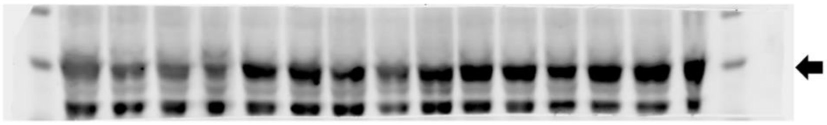

Raw Western Blot Images from Supplementary Figure 4. These raw western blot images were shown in cropped form as the top four panels in Supplementary Figure 4.

Rabbit anti p-Eif2α, 680 nm image

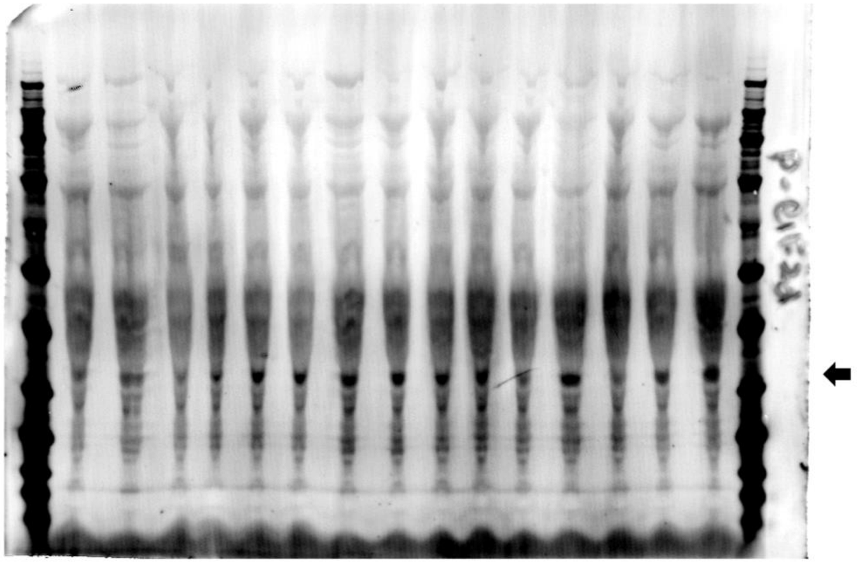

Rabbit anti Eif2α, 680 nm image

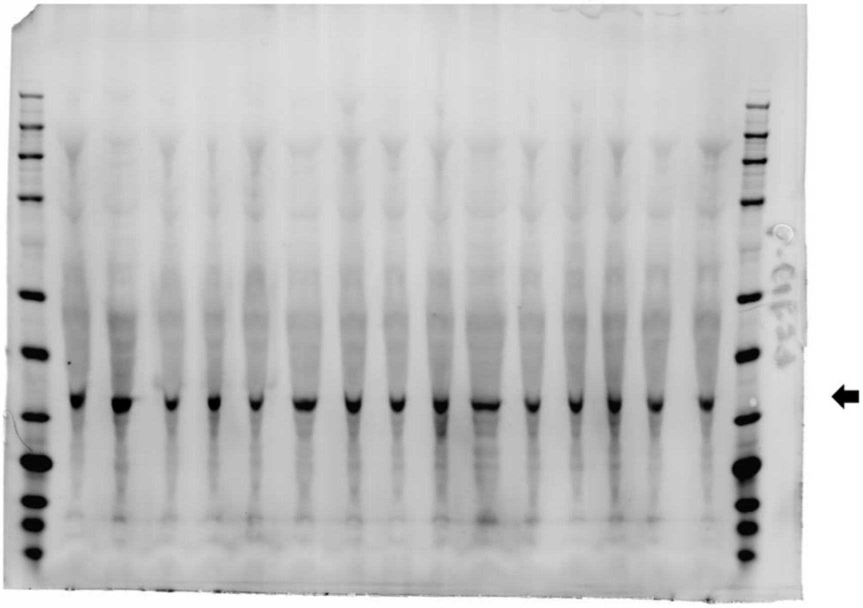

Raw Western Blot Images from Supplementary Figure 4. These raw western blot images were shown in cropped form as the fifth and sixth panels in Supplementary Figure 4.

Rabbit anti p-Eif2α, 680 nm image

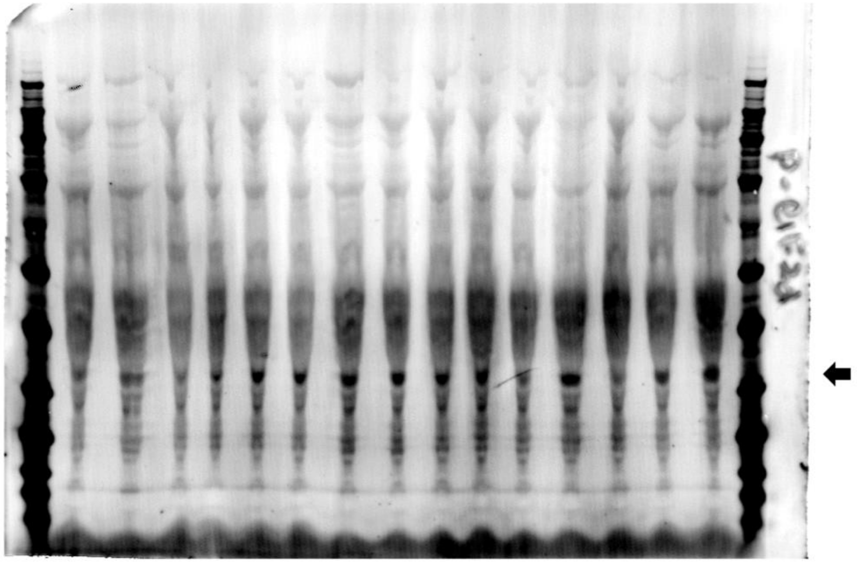

Rabbit anti Eif2α, 680 nm image

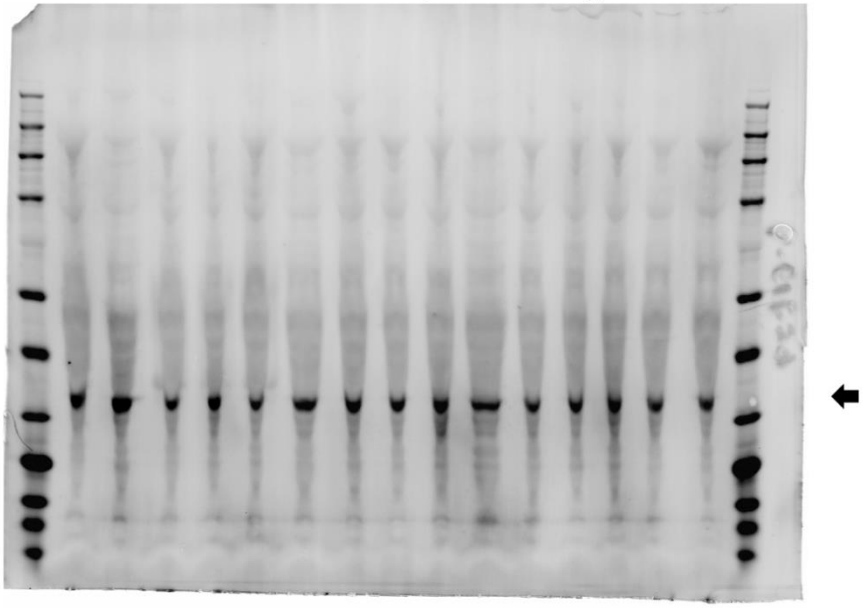

Raw Western Blot Images from Supplementary Figure 4. These raw western blot images were shown in cropped form as the seventh and eighth panels in Supplementary Figure 4.

Rabbit anti Atf4, 800 nm image

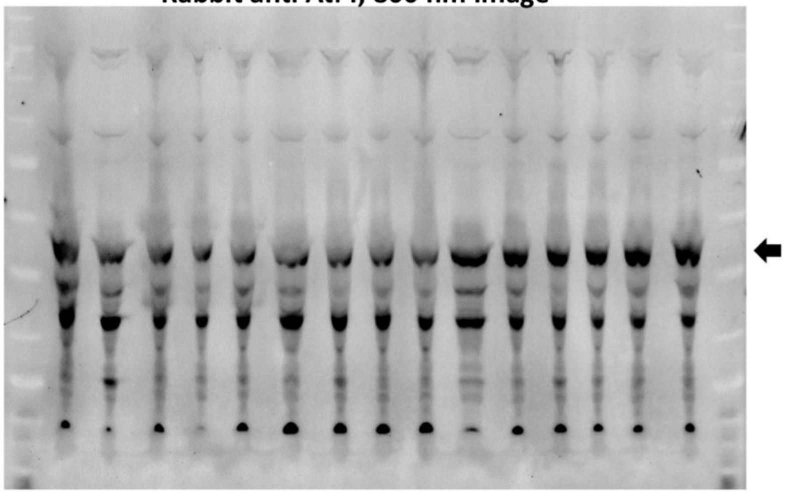

Mouse anti Chop, 680 nm image

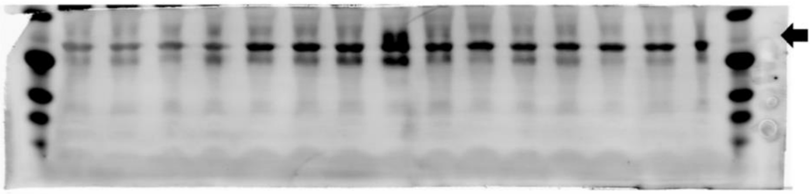

Goat anti Gapdh, 680 nm image

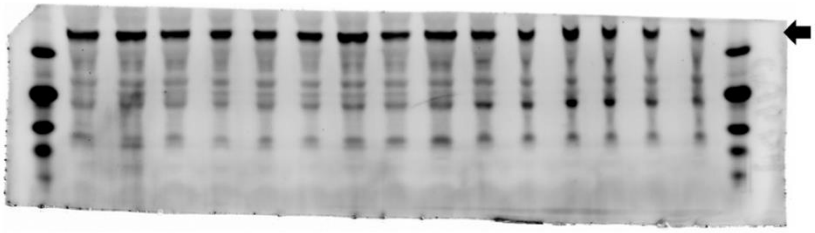

Raw Western Blot Images from Supplementary Figure 4. These raw western blot images were shown in cropped form as the bottom three panels in Supplementary Figure 4.
